# The MYB family and their response to abiotic stress in ginger (*Zingiber officinale* Roscoe)

**DOI:** 10.1186/s12864-024-10392-1

**Published:** 2024-05-11

**Authors:** Hai-Tao Xing, Jia-Yu Shi, Shi-Qing Yin, Qing-Hong Wu, Jian-Ling Lv, Hong-Lei Li

**Affiliations:** 1 College of Landscape Architecture and Life Science/Institute of Special Plants, Chongqing University of Arts and Sciences, Chongqing, 402168 China; 2 Biological Sciences Research Center, Academy for Advanced Interdisciplinary Studies, Southwest University, Chongqing, 400715 China; 3 Chongqing Academy of Chinese Materia Medica, Chongqing, 400065 China; 4 Chongqing Key Laboratory for Germplasm Innovation of Special Aromatic Spice Plants, Chongqing University of Arts and Sciences, Chongqing, 402168 China

**Keywords:** Ginger, ZoMYB, Rhizome development, Abiotic stress, Expression patterns

## Abstract

**Background:**

*Zingiber officinale* Roscoe, colloquially known as ginger, is a crop of significant medicinal and culinary value that frequently encounters adversity stemming from inhospitable environmental conditions. The MYB transcription factors have garnered recognition for their pivotal role in orchestrating a multitude of plant biological pathways. Nevertheless, the enumeration and characterization of the MYBs within *Z. officinale* Roscoe remains unknown. This study embarks on a genome-wide scrutiny of the MYB gene lineage in ginger, with the aim of cataloging all *ZoMYB* genes implicated in the biosynthesis of gingerols and curcuminoids, and elucidating their potential regulatory mechanisms in counteracting abiotic stress, thereby influencing ginger growth and development.

**Results:**

In this study, we identified an MYB gene family comprising 231 members in ginger genome. This ensemble comprises 74 singular-repeat MYBs (1R-MYB), 156 double-repeat MYBs (R2R3-MYB), and a solitary triple-repeat MYB (R1R2R3-MYB). Moreover, a comprehensive analysis encompassing the sequence features, conserved protein motifs, phylogenetic relationships, chromosome location, and gene duplication events of the *ZoMYBs* was conducted. We classified ZoMYBs into 37 groups, congruent with the number of conserved domains and gene structure analysis. Additionally, the expression profiles of *ZoMYBs* during development and under various stresses, including ABA, cold, drought, heat, and salt, were investigated in ginger utilizing both RNA-seq data and qRT-PCR analysis.

**Conclusion:**

This work provides a comprehensive understanding of the MYB family in ginger and lays the foundation for the future investigation of the potential functions of *ZoMYB* genes in ginger growth, development and abiotic stress tolerance of ginger.

**Supplementary Information:**

The online version contains supplementary material available at 10.1186/s12864-024-10392-1.

## Background

The plant *Zingiber officinale* Roscoe, commonly known as ginger, belongs to the *Zingiberaceae* family and plays a significant role in the pharmaceutical and food industries. The therapeutic attributes of ginger are well-documented, encompassing anti-oxidative, anti-inflammatory, and neuroprotective properties [1–3]. The rhizome of ginger, a revered culinary spice and a staple in dietary supplements, boasts a rich composition of essential amino acids, minerals, vitamins, dietary fibers, and flavonoids [4]. The development of the ginger rhizome is a delicately regulated biological process, underpinned by a suite of genes pivotal to its morphogenesis and maturation [5]. Moreover, with the release of the ginger genome sequence [6], has spurred explorations into transcription factor (TF) families, such as AP2/ERF and GRAS, posited to influence rhizome ontogeny [5, 7]. Despite these advances, such factors constitute but a fraction of the elaborate transcriptional network governing rhizome development in ginger.

MYB transcription factors, one of the most expansive and functionally versatile TF families in eukaryotes, are characterized by a conserved MYB DNA-binding domain (DBD) at the N-terminus, typically composed of one to four imperfect repeats (R). These R units, each spanning 51-52 amino acids, form a helix-turn-helix (HTH) structural motif [8–10]. MYB proteins are stratified into four subfamilies based on the number of R repeats: MYB-related (1R-MYB, featuring a single or partial R repeat), R2R3-MYB, R1R2R3-MYB (encompassing three R repeats), and the less understood 4R-MYB proteins. The C-terminal region of MYB proteins is notably divergent, reflecting the family's broad regulatory spectrum [11–13]. The R2R3-MYB subfamily, distinguished by two contiguous R domains, is recognized as the most populous and functionally significant within the MYB constellation. Evolutionary theories suggest that the R2R3-MYB lineage may have evolved following the loss of the R1 repeat or, as posited by Jiang et al. (2004), through an intragenic duplication event that appended the R1 repeat to pre-existing R2R3 MYB genes [14, 15]. Conversely, the 4R-MYB subfamily, the smallest contingent, remains enigmatic in its functional roles [13].

MYB TFs exhibit a widespread distribution in higher plants. Prolific research has illuminated their regulatory involvement in primary and secondary metabolism, as well as cell morphogenesis [16, 17]. Misra et al., (2010) illustrated that the ectopic overexpression of *AtMYB12* in tobacco resulted in enhanced resistance to insect pests *Spodoptera litura* and *Helicoverpa armigera*, attributed to elevated rutin levels [18]. Similarly, *AtMYB11*, *AtMYB12*, and *AtMYB111* have been shown to individually augment genes within the phenylpropanoid pathway, such as those encoding flavanone 3-hydroxylase (F3H), chalcone isomerase (CHI), chalcone synthase (CHS) and flavonol synthase (FLS) proteins, thereby amplifying flavonol biosynthesis [18–21]. In *Epimedium sagittatum*, the R2R3-MYB regulator MYBF1 was found to activate the *EsF3H* (flavanone 3-hydroxylase) and *EsFLS* (flavonol synthase) promoters, with its overexpression in tobacco leading to an increase in flavonol and a decrease in anthocyanin levels in flowers [22]. In *Malus domestica,* the MYB factors *MdMYBA*, *MdMYB1*, and *MdMYB3* have been identified as key determinants in the biosynthesis of red-pigmented anthocyanins in apple peel [23–25]. Moreover, MYB TFs have been implicated in the secondary metabolism of lignin. In Arabidopsis, *SND1*, associated with the cell wall formation, directly targets *MYB83* and *MYB46*, inducing their expression, which in turn activates *MYB58*, *MYB63*, and *MYB85*. These MYBs interact with AC elements in the promoters of lignin biosynthesis genes, driving their expression [26–28]. Chen et al., (2017) highlighted the role of Rep-MYBs, a repressor-type R1R2R3-MYB, in inhibiting G2/M-specific genes in response to DNA damage in Arabidopsis [29].

There is growing evidence indicating that MYB TF also has a crucial function in plant responses to abiotic stress [30]. In Arabidopsis, *AtMYB2* is induced by dehydration, salt stress, and exogenous ABA, with its overexpression in transgenic plants leading to the up regulation of drought-responsive genes *RD22* and *ADH1*, suggesting a role in ABA-mediated drought stress responses [31]. Wyrzykowska et al., (2022) identified *MYB33*, *MYB65*, and *MYB101* as key players in the Arabidopsis drought response via the ABA signaling pathway [32]. *AtMYB2* is also implicated in salt stress tolerance, as evidenced by the enhanced resistance of *AtMYB2-OE* (Overexpression of *AtMYB2*) lines to salinity [33]. Furthermore, increased AtMYB44 levels have been associated with improved salt stress resistance [34]. The induction of *AtMYB41* and *AtMYB96* by drought, ABA, and salt stress in Arabidopsis [35, 36], the overexpression of *OsMYB3R-2* in rice enhancing resistance to freezing, drought, and salinity [37], and the response of *AtMYB68* to high temperatures [38] collectively underscore the multifaceted role of MYB TFs in stress adaptation. *OsMYB55* has been observed to bolster amino acid metabolism, contributing to high-temperature tolerance in rice [39].

Ginger, with its substantial economic importance as a medicinal and culinary crop, is often challenged by abiotic stresses such as extreme temperatures, salinity, and drought, which hinder rhizome cultivation and reduce yields [40, 41]. The identification of MYB as a master regulator of multiple pathways in plants has been established. However, despite its identification in numerous plant species, comprehensive cataloging of MYB in *Z. officinale* Roscoe has not been conducted. The successful sequencing of the ginger genome has enabled the genomic analysis of ginger MYB genes [6]. This study aims to delineate the MYB gene family in ginger by identifying the signature MYB repeats, cataloguing candidate genes, and probing their coding proteins in ginger databases. Additionally, we have embarked on an analysis of the evolutionary and expression dynamics of the MYB family in ginger, with the anticipation that our findings will enrich our understanding of MYB genes' roles in this economically significant plant.

## Materials and methods

### Gene identification and classification

In light of the genomic data from our ginger genome project, previous methodologies have elucidated the presence of a copious MYB gene repertoire within the *Zingiber* genome [6]. Candidate genes were selected using BLASTP, with a score value of ≥100 and e-value of ≤ e-10. The MYB domain HMM profile (PF00249) was obtained from the Pfam protein family database (http://pfam.xfam.org/). HMMER 3.1 was used to perform a HMM search against the *Z. officinale* genome database. A manual assessment was conducted using the Pfam database (http://pfam.janelia.org/) to verify all the putative candidate MYB genes.

Arabidopsis MYB protein sequences were obtained from the TAIR database (https://www.arabidopsis.org/). The derived MYB proteins from ginger and those of Arabidopsis were aligned using MAFFT version 7 with default parameters [42], followed by manual excision of non-homologous regions within the alignment.. A maximum likelihood (ML) phylogenetic analysis was conducted using FastTree with the JTT+G substitution model, incorporating 1000 non-parametric bootstrap (BS) replicates. Gaps within the data were treated as missing information [43, 44].

### Sequence analysis

Physicochemical properties of each MYB gene, including open reading frame (ORF) length, protein length, exon-intron distribution, isoelectric point (pI), and molecular weight (MW) for were analyzed using online ExPASy (http://www.expasy.ch/tools/pi_tool.html). In addition, we determined the conserved motifs of the MYB proteins from ginger, using the MEME (http://meme-suite.org/) program.

### Chromosomal distribution and duplication of ZoMYB genes

The physical distribution map of *ZoMYB* genes on chromosomes was generated according to position information provided by the ginger genome [6]. Gene duplication events of *ZoMYBs* were analysed by MCScanX. The MYB syntenic relationship between ginger and *Arabidopsis thaliana*, *Solanum tuberosum*, *Musa acuminata*, and *Oryza sativa* were determined by Dual Systeny Plotter software of TBtools [45]. Non-synonymous (Ka) and synonymous (Ks) substitution rates were calculated using the KaKs_Calculator (http://code.google.com/p/kaks-calculator/wiki/KaKs_Calculator) [46].

### Promoter cis-regulatory element analysis

The 2000 bp upstream regions of the start codon of *ZoMYB* genes were obtained from the *Z. officinale* Roscoe genome. *cis*-acting regulatory elements within the ZoMYB genes were identified using the online tool PlantCARE (http://bioinformatics.psb.ugent.be/webtools/plantcare/html/). The outcomes were visualized using the Simple biosequence view feature of TBtools [45].

### Plant materials

The ginger cultivar ‘LAIWU No.2’ were used in this study. Seedlings approximately six months of age, including leaves (third position from the apical to basal stem), roots, leaf buds, rhizome buds, flower buds, mature flowers, basal stems, flower petioles, 1^st^, 2^nd^, and 3^rd^ rhizome inter-nodes,were collected for the expression analyses of *ZoMYB* genes. In order to examine the role of ZoMYB genes in response to various abiotic stress responses, two-month-old seedlings underwent drought and salt stress treatments. The seedlings were exposed to a 15% PEG6000 solution and a 200 mM NaCl solution, respectively, for watering purposes. A 0.1 mM ABA solution was sprayed onto ginger leaves. Heat and cold stress treatments were administered at 40°C and 4°C, respectively. Leaf samples were harvested at 0, 1, 3, 6, 12, 24, and 48 hours post cold, drought, and salt treatments, respectively. Each response was replicated thrice. For heat treatment, leaf samples were collected at 0, 1, 3, 6, 12, and 24 hours. Collected samples were immediately frozen in liquid nitrogen and stored at −80°C.

### Subcellular location analyses

The subcellular localization of ZoMYBs were predicted using the WoLF PSORT online web server (https://wolfpsort.hgc.jp/). To confirm the location of ZoMYB#188 and ZoMYB#149, their fulllength coding sequence (CDS) without stop codon were cloned into pCAMBIA1300 and fused at the C-terminal of the green fluorescent protein (GFP) using the primer pairs CDS-BamH I -Faw/noTAA-BamH I-Rev, respectively. The resulting ZoMYB-GFP fusion expression vectors were sequenced and introduced into Agrobacterium GV3101 using the freeze–thaw method. The vector GV3101 (*A. tumefaciens*) carrying *35S::GFP-ZoMYB* and a control vector (*35S::GFP*) were then infiltrated into tobacco epidermal leaves. Green fluorescent protein (GFP) fluorescence driven by the 35S promoter was observed 36–48 h post-infiltration using a confocal microscope (Olympus FV1200). Plant nuclei were visualized by staining with 4, 6-diamidino-2- phenylindole (DAPI).

### Expression analysis of ZoMYB genes by RNA-seq and qRT-PCR

RNA-seq was performed on samples acquired 12 h post ABA, heat, cold, salt, and drought treatments.The transcriptome data were deposited in the NCBI Short Read Archive (Project Accession Number: SRP064226). Expression levels in RNA-Seq were analyzed using FPKM values. TBtools was used to transform the FPKM values to log_2_ (FPKM + 1) values and generate an expression heatmap for the *ZoMYB* genes [45]. Total RNA was extracted from collected samples using the Plant RNeasy Mini Kit (Qiagen). Subsequently, cDNA was synthesized utilizing the PrimeScript RT reagent Kit with gDNA Eraser (Takara, Dalian, China). Quantitative reverse transcription polymerase chain reaction (RT-qPCR) was conducted on a TP800 Thermal Cycler Dice Real Time System (Takara) using SYBR Premix ExTaq (Takara, Kyoto, Japan). The total reaction volume was 10 μL, comprising 5 μL of SYBR mix, 0.4 μL of primer mix (10 μM), 0.5 μL cDNA template, and 4.1 μL ddH_2_O. The qRT-PCR amplification conditions were as follows: 95◦C for 1 min, followed by 40 cycles at 95◦C for 10 s and 60◦C for 30 s. A melting curve ranging from 60 to 95◦C was generated to verify primer specificity. The *ZoTUB2* gene as the internal reference. Three biological replicate experiments were conducted. Relative expression levels of *ZoMYBs* were determined using the 2^−ΔΔ CT^ method [47]. qRTPCR primers were designed using DNAman software (Supplementary Table 9).

## Results

### Comprehensive identification of ZoMYB gene family in ginger

A total of 231 *ZoMYBs* were identified in *Z. officinale* after removing redundant repetitive sequences. These genes, now designated *ZoMYB#1* through *ZoMYB#231*, were chromosomally mapped and systematically renamed in accordance with their chromosomal positioning. The ZoMYB family is categorized into three distinct subfamilies: 156 R2R3-MYB proteins (2R-MYB sub family), 1 R1R2R3-MYB protein (3R MYB subfamily), and 74 MYB-related proteins (MYB related subfamily). Notably absent were representatives of the 4R-MYB class. Detailed attributes including coding sequence, protein sequence, sub-cellular localization, isoelectric point (PI), and molecular weights (MWs) are meticulously compiled in Supplementary Tables S1. *ZoMYB#49* (*Maker00069396*) encodes the largest protein sequence with 1649 amino acid (aa), while *ZoMYB#97* (*Maker00078016*) encodes the most compact protein at 73 aa, reflective of its singular MYB DBD domain. The molecular weights of the proteins span from 8243.9 Da (ZoMYB#97) to 182,772.28 Da (ZoMYB#49), with isoelectric points ranging from 4.38 (ZoMYB#97) to 9.96 (ZoMYB#215), respectively.

### Phylogenetic insights into MYB genes in ginger and Arabidopsis

**Fig. 1:**
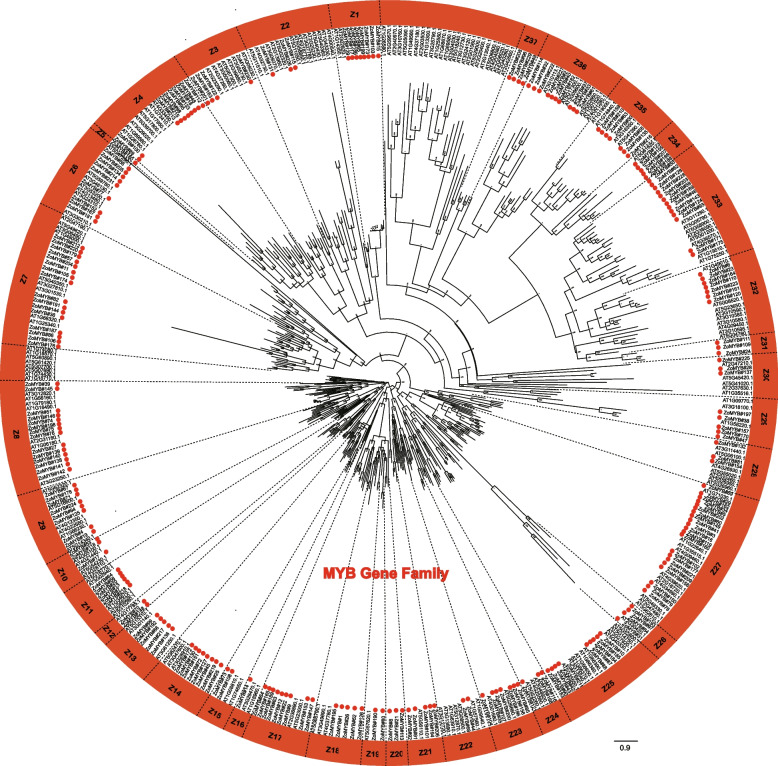
Phylogenetic analysis of MYB proteins from ginger and Arabidopsis. Node values indicate bootstrap support for each branch. Proteins prefixed with "Zo" denote *Z. officinale*”origin and are highlighted with red dot. The 37 ZoMYB subgroups are encircled in red (Z1-Z37)

A phylogenetic reconstruction was undertaken using the amino acid sequences of the full complement of 231 ZoMYB and 197 AtMYB proteins to elucidate evolutionary relationships. Based on the phylogenetic tree, 231*ZoMYB* genes in ginger were divided into 37 groups (Z1-Z37) (Fig. 1, Supplemetary Document S1). Relying on the group classification of R2R3-MYB in Arabidopsis, the R2R3-MYBs were mainly belong to groups, designated Z2, Z3, Z6, Z7, Z8, Z9, Z10, Z11, Z12, Z13, Z14, Z15, Z17, Z18, Z20, Z21, Z22, Z23, Z24, Z25, Z27, Z28, Z32, Z33, and Z34. The MYB-related genes were mainly belong to Z1, Z26, Z29, Z30, Z31, Z35, Z36, and Z37. Notably, clades Z7, Z9, Z13, Z14, Z18, Z19, Z21, Z22, Z23, Z25, Z31, and Z32 have both MYB-related and R2R3-MYB genes. For example, clade Z6 included two MYB related type proteins, ZoMYB#56 and ZoMYB#77, together with other 7 R2R3 MYB proteins (ZoMYB#134, #152, #167, #181, #205, #231, and #214). Clade Z27, with 21 members, emerged as the most populous, while clades Z5 and Z16 were the least diverse, each containing a single membe.

### Divergence in gene structure and motif composition within ZoMYBs

**Fig. 2:**
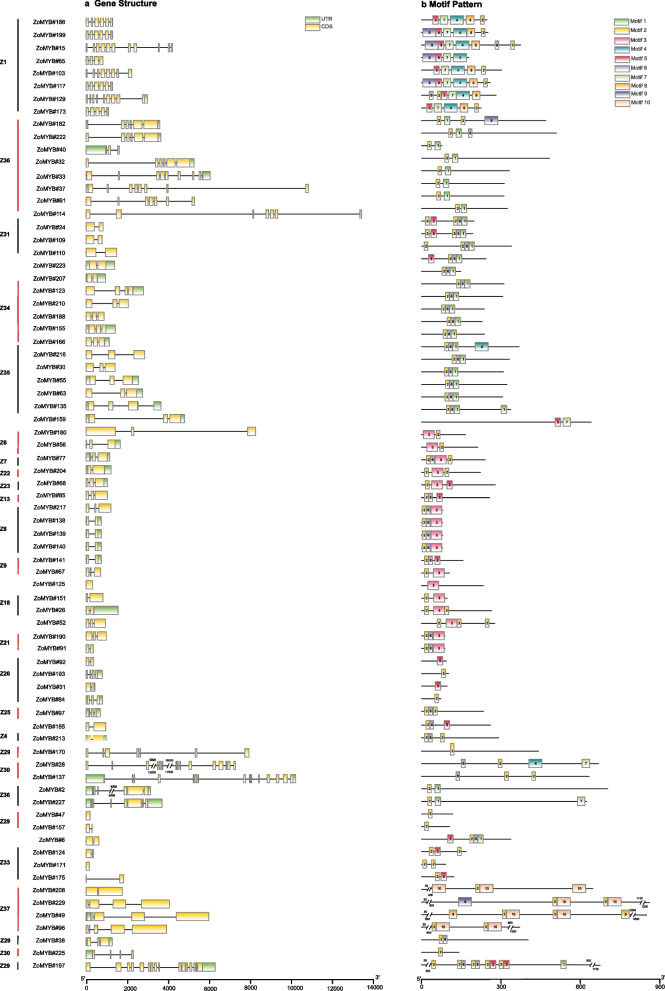
Distributions of gene structure and conserved motifs in 1R-*ZoMYB* and 3R-*ZoMYB* genes. **a** Exon/Intron structures of 1R-and 3R-*ZoMYB* genes. Green boxes represent untranslated regions (UTRs), yellow boxes denote exons, and black lines indicate introns. **b** Conserved motifs in 1R-and 3R-ZoMYB proteins, numbered 1–10 and depicted in various colors, with sequence details provided in Supplementary Figure S1. Scale at the bottom approximates protein length

An intricate examination of the exon–intron architecture of ZoMYB transcription factors (TFs) in ginger was undertaken to elucidate the sequence diversity inherent to this gene family. The sequence structure of 1R-MYB, R1R2R3-MYB and R2R3-MYB in ginger were demonstrated separately. Illustrated in Fig. 2a, the 74 genes of the 1R-MYB category alongside a singular R1R2R3-MYB gene exhibited a range of exon counts, extending from a minimum of two to a maximum of 19. The intricacies of this exon–intron structural variance among the 74 1R-MYB genes, as well as the sole R1R2R3-MYB gene, are depicted in Fig. 2a. The exon tally for the 1R-MYB genes varied from one to 16, with the lone R1R2R3-MYB gene (*ZoMYB#197*) comprising a total of 19 exons. A notable proportion of the 1R-MYB genes predominantly possessed two (25.68%, 19/74) or three exons (29.73%, 22/74), while configurations of 10, 11, 15, and 16 exons were unique occurrences. As delineated in Fig. 4a, the ensemble of 156 R2R3-MYB genes was characterized by a disparity in exon numbers, which spanned from one to 11. A majority of the R2R3-MYBs in ginger conformed to the canonical splicing pattern of three exons (60.26%, 94 of 156 R2R3-MYB) interspersed by two introns (20.51%, 32/156). The CDS of most *ZoMYB* genes were interrupted by introns, with the exception of six *ZoMYBs* genes (*ZoMYB#47, ZoMYB#122, ZoMYB#151, ZoMYB#163, ZoMYB#171, ZoMYB#201*) which remained uninterrupted. In addition, when viewed in conjunction with the phylogenetic tree classification, members of the *ZoMYB* gene family that clustered within the same phylogenetic clade generally shared similar or identical exon counts, notwithstanding variations in exon positioning. For instance, the *ZoMYB* gene groups Z19 and Z20 both contained three exons, whereas the Z8 clade exhibited a range of two to six exons (Supplementary Table S1). In sum, the exon numbers of *ZoMYB* genes from ginger are quite divergent, however, the closer phylogenetical relationship is indicative of a greater homogeneity in sequence structure.

**Fig. 3:**
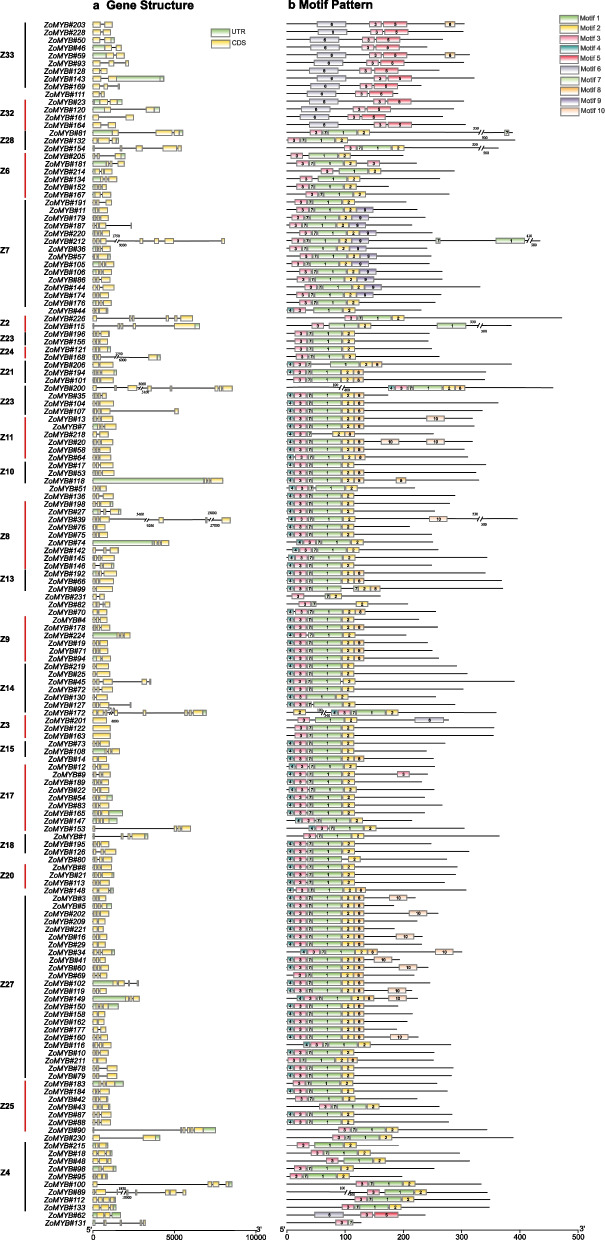
Distributions of gene structure and conserved motifs in R2R3-*ZoMYB* genes. **a** Exon/Intron structures of R2R3-*ZoMYB* genes. Green boxes, yellow boxes, and black lines indicate the 5′- and 3′-untranslated regions (UTR), exons, and introns, respectively. **b** Conserved motifs of R2R3-*ZoMYB* proteins. The motifs, numbers 1–10, denoted by a different color, is accompanied by sequence information in Supplementary Figure S3. The protein length can be estimated using the scale at the bottom

**Fig. 4:**
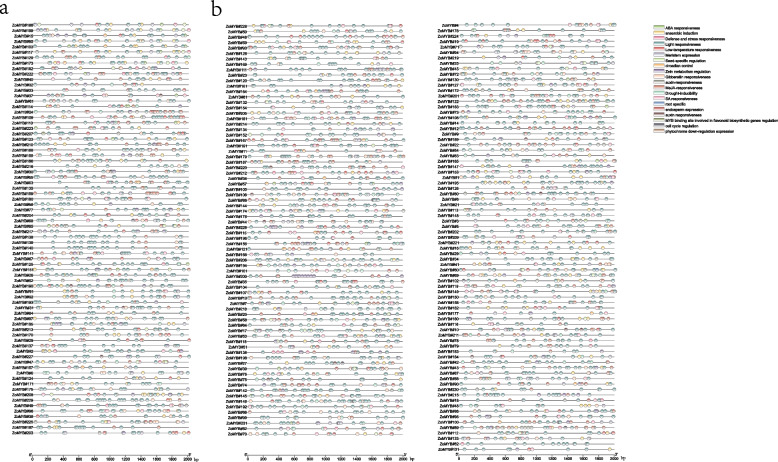
Regulatory elements in the promoter of *ZoMYBs*. **a** Regulatory elements in 1R and 3R-MYB genes promoter. **b** Regulatory elements in R2-R3 MYB genes promoter, with Different colors denoting different *cis*-acting elements

To illuminate the conserved domains within ZoMYB proteins, motif analysis via MEME suite was performed, identifying twenty motifs across the 1R-MYB, R1R2R3-MYB and R2R3-MYB proteins of ginger (Figs. 3b, and 4b). As shown in Fig. 3b and Supplementary Figure S2, motif 2 and motif 6 were ubiquitous across the 1R-MYB proteins, whereas motif 1, 2, 3, and 7 were integral in encoding the MYB DNA-binding domain (DBD) within the R2R3-MYB proteins. In 1R-MYB types, different groups had different motifs, and motif 2 was common in all 1R-MYB proteins in Fig. 3b. The members from groups Z1 group were characterized by the presence of 9, 5, 7, 4, 8 and 2, while groups Z34 and Z35 harbored motifs 2, 6, and 1. Group Z31 was distinguished by motifs 5, 2, 6, and 1, and group Z8 by motifs 2, 6, and 3. This motif distribution underscores the lower sequence similarity among 1R-MYB proteins across different phylogenetic clades. Within the subset of 156 R2R3-MYB proteins, 85 were found to contain motifs 4, 3, 7, 1, and 2. ZoMYB#131 was exclusively composed of motifs 3 and 7. A unique motif signature, comprising motifs 6, 3, and 5, was identified in the R2R3-MYB proteins associated with the Z33 and Z32 clades. The 3R-MYB (ZoMYB#197) has three conserved motifs 2, 5 and 6, with motifs 2 repeated four times, and motifs 6 and 5 each being duplicated within this protein structure. Moreover, specific motif patterns unique to ZoMYB were discerned. For instance, ZoMYB#31 and ZoMYB#84 were solely characterized by motifs 2 and 5, respectively.

### Analysis of cis-acting elements of ZoMYB promoters

A total of 57 *cis*-acting elements were identified in the promoter region (upstream 2000 base pair) of *ZoMYBs*, which could be classified into five categories: phytohormones (ABA responsiveness elements, Auxin responsiveness elements, Gibberellin responsiveness elements, MeJA responsiveness elements, SA responsiveness elements), environmental stress (Drought-inducibility elements, Defense and stress responsiveness elements, Low-temperature responsiveness elements, Anaerobic induction elements), photo-responsive elements (Light responsiveness elements, phytochrome down-regulation expression elements), growth and developmental elements (Cell cycle regulation elements, Circadian control elements, Root specific elements, Seed-specific regulation elements, Endosperm expression elements, Meristem expression elements) and secondary metabolic elements (MYB binding site involved in flavonoid biosynthetic genes regulation, Zein metabolism regulation), as delineated in Fig. 4, Supplementary Table S2, and Supplementary Table S3. The majority of *ZoMYB* genes exhibite the presence of a minimum of one phytohormone-responsive element within their promoter regions. A total of twelve phytohormone-responsive *cis*-acting elements were discerned, including ABRE, AuxRR-core, TGA-element, TGA-box, P-box, GARE-motif, TATC-box, TGACG-motif, CGTCA-motif, TCA-element, SARE, and O2-site. The *cis*-acting elements play a crucial role in regulating the responsiveness to ABA, auxin, gibberellin, methyl jasmonate, salicylic acid, and zein metabolism. Meanwhile, the ABRE responsiveness elements were the most common in the *ZoMYB* gene promoters. Furthermore, 34 *cis*-acting elements play a role in light responsiveness. Eight *cis*-regulatory elements associated with response to abiotic stresses were identified, including the low-temperature responsive element (LTR), anaerobic induction elements (ARE), drought-inducibility element (MBS), defense and stress responsive element (TC-rich repeats), and circadian control element (circadian).The Light responsiveness elements (2873), MeJA-responsiveness elements (934), ABA responsiveness elements (574), Anaerobic induction elements (351), Gibberellin responsiveness elements (228), Drought-inducibility elements (185) was observed in the majority of the 1R-and 2R-*ZoMYB* genes. The 3R-*ZoMYB* gene (*ZoMYB#197*) is notably enriched with light-responsive elements (15 in total) and possesses a modest array of stress-responsive elements, including two MeJA-responsiveness elements, two ABA responsiveness elements, and two anaerobic induction elements. The MSA-like element, associated with cell cycle, was uniquely present in 10 *ZoMYB* genes (*ZoMYB#220*, *ZoMYB#44*, *ZoMYB#154*, *ZoMYB#89*, *ZoMYB#18*, *ZoMYB#102*, *ZoMYB#68*, *ZoMYB#27*, *ZoMYB#26*, *ZoMYB#74*)*.* A quartet of meristem expression elements were identified in each of *ZoMYB#186*, *ZoMYB#133*, and *ZoMYB#25*. Furthermore, a singular MYB binding site, integral to the regulation of flavonoid biosynthetic gene elements, was found within *ZoMYB#205*, *ZoMYB#124*, *ZoMYB#207*, *ZoMYB#147*, *ZoMYB#88*, *ZoMYB#117*, *ZoMYB#65*, *ZoMYB#132*, *ZoMYB#48*, *ZoMYB#24*.

### Chromosomal distribution, gene duplication, and synteny analyses of ZoMYB genes

**Fig. 5:**
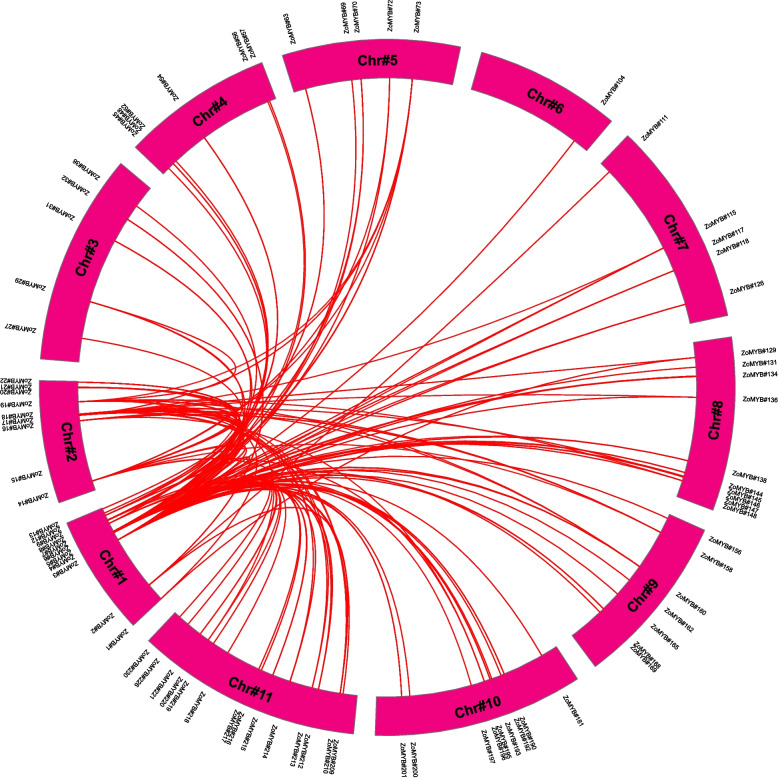
A schematic representation of the inter-chromosomal relationships of ginger’s *MYB* genes. Red lines indicate duplicated *ZoMYB* gene pairs. The chromosome number is indicated in the middle of each bend bar

Chromosomal allocation studies revealed a heterogeneous dispersal of *ZoMYB* genes across the 11 chromosomes of ginger (Supplementary Figure S4). Chromosomes 10, 6, and 8 harbored the highest contingents of *ZoMYB* (29, 27, and 27 genes, respectively), whereas chromosome 2 presented with a minimal assemblage of 9 *ZoMYB* genes. Proliferation of the *ZoMYB* gene family has predominantly been propelled by gene duplication events. It was found that 34 *ZoMYB* genes are clustered into 15 tandem duplication regions among ginger chromosomes 4, 5, 6, 8, 9, 10, and 11(Supplementary Figure S4 and Supplementary Table S4). Three tandem-duplicate gene pairs were found on chromosome 6, (*ZoMYB#87-ZoMYB#88*, *ZoMYB#93-ZoMYB#94*, *ZoMYB#89-ZoMYB#90*) and chromosome 10 (*ZoMYB#188-ZoMYB#189*, *ZoMYB#184-ZoMYB#185*, *ZoMYB#193-ZoMYB#194*); Additionally, two tandem-duplicate gene pairs were detected on chromosome 5 (*ZoMYB#66-ZoMYB#67, ZoMYB#75-ZoMYB#76*), chromosome 8 (*ZoMYB#147-ZoMYB#148*, *ZoMYB#138-ZoMYB#139-ZoMYB#140*), chromosome 9 (*ZoMYB#165-ZoMYB#166*, *ZoMYB#170-ZoMYB#171*) and chromosome 11 (*ZoMYB#225-ZoMYB#26*, *ZoMYB#210-ZoMYB#211*), with a sole tandem-duplicate gene pair on chromosome 4 (*ZoMYB#42-ZoMYB#43*). Among these tandem duplicated gene pairs, eight exhibited identical (*ZoMYB#66-ZoMYB#67*, *ZoMYB#75-ZoMYB#76*) or homologous (*ZoMYB#89-ZoMYB#90*, *ZoMYB#42-ZoMYB#43*, *ZoMYB#138-ZoMYB#139-ZoMYB#140*, *ZoMYB#147-ZoMYB#148*, *ZoMYB#87-ZoMYB#88*, *ZoMYB#184-ZoMYB#185*) motifs. And the remaining seven pairs contained different motifs (*ZoMYB#93-ZoMYB#94*, *ZoMYB#165-ZoMYB#166*, *ZoMYB#170-ZoMYB#171*, *ZoMYB#188-ZoMYB#189*, *ZoMYB#193-ZoMYB#194*, *ZoMYB#225-ZoMYB#226*, *ZoMYB#210-ZoMYB#211*). Beyond tandem duplications, 82 pairs of segmentally duplications were found within the ginger genome (Supplementary Table S4). The synteny analysis highlighted a conservation of MYB transcription factors across ginger chromosomes, with numerous homologous genes situated on disparate chromosomal tracts (Fig. 5).

### Evolutionary analysis of ZoMYB genes

**Fig. 6:**
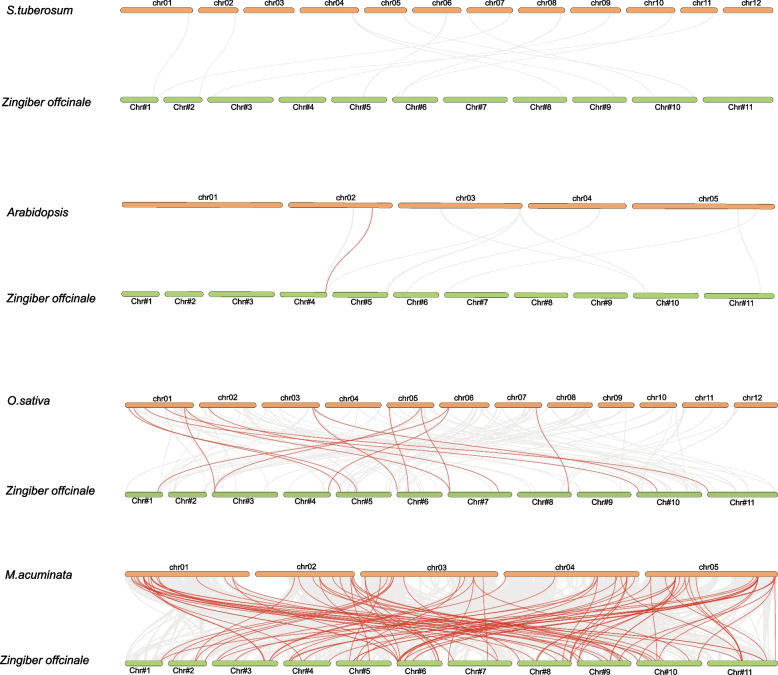
Synteny analysis of *MYB* genes between ginger and other four representative plant species. Gray lines in the background represent collinear blocks, while red lines indicate syntenic *MYB* gene pairs

In order to gain a deeper understanding of the phylogeny of the MYB family, we conducted a comprehensive synteny analysis comparing the ginger genome with those of four divergent plant species, including two monocots (*M.acuminata* and *O.sativa*), and two dicots (*A. thaliana* and *S. tuberosum*). The analysis revealed that a total of 75 *ZoMYB* genes exhibit syntenic relationships with 91 corresponding genes in *M.acuminata*, This was followed by 14 syntenic relationships with *O.sativa*, and a single syntenic relationship with an *A. thaliana* gene (Fig. 6 and Supplementary Table S5). There were 91, 15, and 1 pairs of orthologous genes identified between ginger and *M. acuminata*, *O. sativa*, and *A*. *thaliana*, respectively. *ZoMYB#153* (*Maker00037045*) was found to be syntenic with three gene pairs in *M.acuminata,* while *ZoMYB#216* (*Maker00052236*) corresponded with four M. acuminata gene pairs*.* The *ZoMYB* genes demonstrated a significant degree of orthology with the reference genomes, with *M.acuminata* demonstrated a significant degree of orthology with the reference genomes, following by *O.sativa* which displayed 15 orthologous gene pairs scattered across chromosomes 1, 2, 3, 5, and 7, and *A. thaliana*, with a single orthologous gene pair on chromosome 2. However, no syntenic relationships were identified between ginger and *S. tuberosum* (Fig. 6 and Supplementary Table S5). Further examination of the syntenic relationships between ginger and *M. acuminata MYB* genes revealed that 10 *ZoMYBs* were linked to two syntenic gene pairs each, 2 *ZoMYBs* were identified to be associated with 3 syntenic gene pairs each, and 1 *ZoMYB* gene was linked to 4 syntenic gene pairs.

To enrich our comprehension of the evolutionary pressures imposed upon the MYB gene lineage, an analysis of the nonsynonymous to synonymous substitution rate ratio (Ka/Ks) for MYB gene pairs was undertaken. As delineated in Supplementary Table S6, it was observed that the Ka/Ks ratios for all tandemly and segmentally duplicated *ZoMYB* gene pairs, as well as the majority of orthologous *MYB* gene pairs, were discerned to be less than one, indicative of purifying selection acting upon these sequences.

### Expression profiling of ginger ZoMYB genes in different tissues

**Fig. 7:**
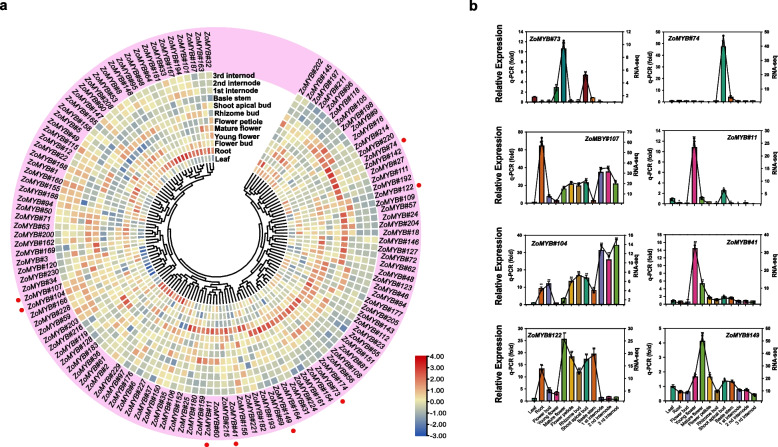
*ZoMYB* gene expression profile analysis in ginger. **a** Hierarchical cluster analysis of *ZoMYB* gene expression profiles in 12 different tissues and developmental stages of ginger as determined by RNA-seq. The red circles represent the randomly selected *ZoMYB* genes that were randomly selected for validation of their expression profiles by qRT-PCR. **b** The expression levels of 8 *ZoMYB* genes were analyzed in 12 samples using qRT-PCR and RNA-seq. The X-axis represents the various tissues, while the Y-axis displays the q-PCR fold changes(vertical bar) and FPKM values(line and scatter) of the candidate genes on the left and right sides, respectively. Data are normalized to the *Tub-2* gene, vertical bars are standard bar. *n* = 3. Mean values and standard deviations (SDs) were obtained from three biological and three technical replicates. The error bars indicate standard deviation. **P < 0.01 and *P < 0.05

To explore the potential function of the *ZoMYB* genes at different stages of ginger development, RNA-sequencing data were utilized to ascertain their expression profiles (Fig. 7a and Supplementary Table S7). To further validate the reliability of the transcriptome data, qRT-PCR analyses were carried out on 12 representative samples for 12 selected *ZoMYB* genes (Fig. 7b). Among the 231 ZoMYB family genes, three *ZoMYBs* (*ZoMYB#92*, *ZoMYB#94*, *ZoMYB#100*) were not detected in any tested samples, suggesting the possibility of highly specialized spatio-temporal expression patterns that our dataset did not capture, or alternatively, these may represent pseudogenes. Among the 12 samples tested, the expression of 100 *ZoMYB* genes were detected (FPKM > 0), and 26 ZoMYB genes were constitutively expressed (FPKM > 1 in all samples). Eight *ZoMYB* genes (*ZoMYB#98*, *ZoMYB#53*, ZoMYB#208, ZoMYB#213, ZoMYB#145, ZoMYB#167, ZoMYB#101, and *ZoMYB#95*) are preferentially expressed in roots, one gene in flower bud (ZoMYB#12), four in young flower (*ZoMYB#41*, *ZoMYB#11*, *ZoMYB#152*, *ZoMYB#215*), two in mature flower (*ZoMYB#149* and *ZoMYB#73*), three in shoot apical bud (*ZoMYB#74*, *ZoMYB#72* and *ZoMYB#66*), one in leaves (*ZoMYB#227*) shown relative higher expression level than other *ZoMYBs*. The expression pattern of some *ZoMYBs* exhibited divergent tendency during different stage of development. For example, the expression levels of *ZoMYB#143/182/155/202* were gradually increased, whereas that of *ZoMYB#34/230/61/161*/*22/23/158/162* were gradually decreased during the rhizome development stages (Fig. 7a). Eight *ZoMYBs* were randomly selected to validate RNA-seq result by qRT-PCR. The results showed that the results are consistent with the RNA-seq (Fig. 7b).

### Expression profiles of ZoMYB genes under abiotic stress conditions

**Fig. 8:**
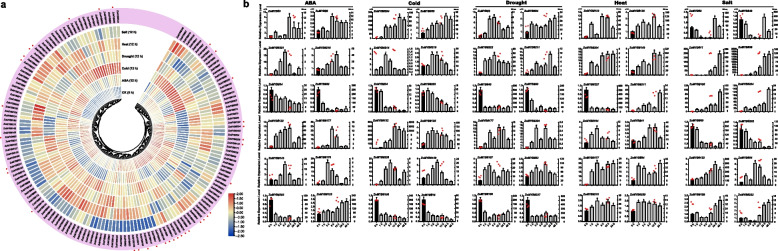
Expression profiles of *ZoMYB* genes under various abiotic stress treatments. **a** *ZoMYB* genes expression in response to various abiotic stress as determined by RNA-seq. Relative expression of different *ZoMYBs* is shown for control and under ABA, cold, drought, heat, and salt stress after 12 h. Red circles indicate the *ZoMYB* genes that were arbitrarily chosen for validation of their stress-induced expression changes through qRT-PCR. **b** The expression of *ZoMYB* genes under abiotic stresses was analyzed using q-PCR and RNA-seq. The X-axis represents different time points after treatment, while the Y-axis displays qRT-PCR fold changes(left, vertical bar) and Fragments Per Kilobase Million (FPKM) values (right, scatter) of candidate genes. The expression levels were normalized to *TUB-2* gene, and vertical bars indicate ± SD. *n* = 3. Mean values and standard deviations (SDs) were obtained from three biological and three technical replicates. The error bars indicate standard deviation. **P < 0.01 and *P < 0.05

To elucidate the potential roles of *ZoMYB* genes under various abiotic stresses, we analyzed RNA-seq data to determine their transcriptional responses to heat, cold, salt, drought, and ABA treatments. A total of 154 *ZoMYB* genes were induced or reduced in response to at least one stress treatment compared with time 0 (CK) (Fig. 8a). Concisely, exposure to cold stress prompted the upregulation of 39 *ZoMYBs*, drought stress influenced 37, heat stress affected another 37, ABA treatment modulated 48, and saline conditions altered the expression of 62 genes (Supplementary Table S8). A subset of 8, 1, 4, and 9 *ZoMYBs* exhibited unique enhancements in response to ABA, cold, heat, and salt stress, respectively. Notably, drought stress did not uniquely induce any *ZoMYB* gene. A total of 13 *ZoMYBs* exhibited an upregulated expression pattern across all five abiotic stress treatments. (Supplementary Figure S5). Ten of these 13 *ZoMYBs* are R2R3-MYB types, while the remaining three are MYB-related types. These include three genes from the Z34 group, three from Z18 group, and two from Z25 group. *ZoMYB#51* was enhanced in drought, heat and ABA stress treatment. Conversely, 49, 32, 40, 25, and 24 *ZoMYB* members were down-regulated by cold, drought, heat, ABA, and salt treatments, respectively. Ten *ZoMYBs* were reduced in all the five abiotic stress treatment, with four belonging to the Z34 group (R2R3-MYB type) and the Z10 group (MYB-related type). Distinct decreases were observed in 3, 10, 4, 1, and 1 *ZoMYBs* in response to ABA, cold, heat, drought, and salt stress, respectively. (Supplementary Figure S6). Nine *ZoMYBs* were reduced in both drought and heat stress. Among these differentially expressed genes, *ZoMYB#3*, *ZoMYB#177*, *ZoMYB#221*, belong to Z34 group were induced, while four genes, namely *ZoMYB#60*, *ZoMYB#5*, *ZoMYB#105*, and *ZoMYB#202* also belong to Z34. Furthermore, we evaluated the expression patterns of 41 randomly selected *ZoMYB* genes under heat, low temperature, drought, salt and ABA stress conditions using qRT-PCR. Each of the 41 chosen *ZoMYB* genes manifested significant upregulation at one or several time points during stress exposure (as illustrated in Fig. 8b), corroborating the expression trends observed in the RNA-seq analysis.

### *Subcellular localization of *ZoMYB* proteins*

**Fig. 9:**
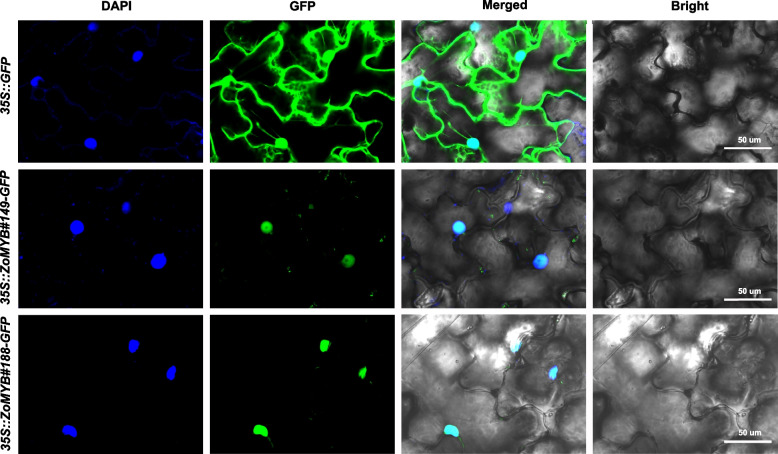
Subcellular localization of seven GFP-fused ZoMYB proteins. Transformation of tobacco epidermal cells with vectors containing *35S::ZoMYB-GFP* constructs or a *35S::GFP* control was followed by fluorescence examination via confocal microscopy at 40 h post-transfection.. Nuclear targeting was confirmed with DAPI staining. Scale bars = 50 μm

The subcellular localization of ginger MYB proteins from ginger was initially predicted using WoLF PSORT (Supplementary Table S1). Among the ZoMYB proteins, a substantial majority of 90.48% (209/231) were predicted to to localize to the nucleus. Additionally, 5.19% (12/231) were predicted to be chloroplastic, 1.30% (3/231) mitochondrial, another 1.30% (3/231) peroxisomal, 0.865% (2/231) cytoplasmic, and the remaining 0.865% (2/231) cytoskeletal. To verify these predictions, two genes, *ZoMYB#149* and *ZoMYB#188*, which exhibited pronounced responsiveness to elevated temperatures and were specifically expressed in certain tissues, were selected for empirical validation through a transient expression assay. The fusion proteins ZoMYB#149-GFP and ZoMYB#188-GFP were observed to accumulated in the nuclei of epidermal cells. This was in stark contrast to the control GFP alone (vector control, *35::GFP*), which was distributed throughout both the cytoplasm and the nucleus of the epidermal cells (Fig. 9). The empirical findings were congruent with the predicted subcellular localizations, reinforcing the accuracy of the initial predictions.

## Discussion

The MYB transcription factor (TF) family constitutes one of the most expansive TF contingents within the plant kingdom. Comprehensive characterizations of the MYB gene clan have been executed across a diverse array of plant species, such as *A. thaliana* [48], *Z. mays* [49], *O. sativa* [48], and *S. tuberosum* [16], wherein these MYB genes have been implicated in an array of physiological processes, encompassing both primary and secondary metabolism, cell cycle regulation, and the response to abiotic and biotic stressors [50–53]. *Z. officinale* Roscoe, a medical and vegetable plants, commands a pivotal role in the pharmaceutical and food industries. Despite the unveiling of the ginger genome in 2021 [6], the exploration of MYB proteins within ginger has remained nascent. Given the pivotal role of MYB proteins within the botanical realm, this study represents the inaugural comprehensive investigation of the MYB gene family predicated on the sequenced genome of *Z. officinale* Roscoe.

In this study, a screening of the ginger genome yielded a total of 231 members, including 156 R2R3-MYBs, 74 1R-MYB proteins, and one R1R2R3-MYB member. However, none of the 4R-MYB members were identified in ginger genome (Supplementary Table S1). The R2R3-MYB subfamily is preeminent within ginger, suggesting that the expansion of this gene family might elucidate the profusion of ZoMYB memebers. Gene duplication events are an important mechanism that results in the evolutionary expansion of gene families. Within the context of ginger, the analysis of gene duplication revealed 15 gene pairs resulting from tandem duplication and 82 pairs resulting segmental duplication. Similarly, in *Casuarina equisetifolia* and *Pyrus bretschneideri*, duplication events have precipitated the expansion of the MYB gene family within their genomes, indicating that tandem and segmental duplication have collectively facilitated the expansion of the MYB gene family across diverse plant species throughout evolutionary history [54, 55]. The Ka/Ks ratios observed amongst the MYB gene pairs indicate that the MYB gene family in ginger may have undergone strong purifying selective pressure during its evolutionary history (Supplementary Table S6).

The physical parameters of MYBs across different plants exhibit considerable. The MWs of the ZoMYB proteins ranged from 8.24 (ZoMYB#97) to 182.77 kDa (ZoMYB#49), with predicted pI values range from 4.38 (ZoMYB#97) to 9.96 (ZoMYB#215). The MW of rice MYB proteins ranged from 7.61 to 170.492 kDa, with pI values from to 3.99 to 12.26, whereas the MW of Arabidopsis MYB (AtMYB) proteins ranged from 7.57 to 158.27 kDa, with pI values from to 4.16 to 10.24 [56]. These findings suggest a conserved evolutionary trajectory for the MYB gene family.

To study the intron distribution in the *Z. officinale* Roscoe genome, we investigated the exon-intron structure of *ZoMYB* genes. Within a given clade, the number of introns and exons was either identical or exhibited similarity. As shown in Figs 2a and 3a, a substantial 93.40%(225/231) of *ZoMYB* genes were disrupted by introns, with the majority harboring between one to eighteen introns. Among the *ZoMYB* genes analyzed, it was found that 22.51% (52/231) and 50.22% (116/231) possessed two or three exons, respectively. This feature of MYB gene structures was similar to the numbers of introns in Arabidopsis and rice [56].

Gene duplication not only augments gene families but also fosters functional diversification. In the context of ginger, the analysis of gene duplication revealed 15 gene pairs stemming from tandem duplications and 82 pairs from segmental duplications. This suggests that ZoMYB has undergone gene expansion due to genomic duplications (Figure 5). The Ka/Ks ratios observed amongst the MYB gene pairs indicate that the MYB gene family in ginger may have been subject to intense purifying selective pressures.

Notably, *ZoMYB#3/4/5* were found to be associated with 26, 14, and 13 segmental duplication gene pairs, respectively, indicating their potential conservation and antiquity. The expression of numerous duplicated ZoMYBs across different tissues suggests that these genes may have redundant or specific cellular functions during the growth and development of ginger. The expression patterns of *ZoMYB#89* and *ZoMYB#90* provide evidence for potential differences between duplicated gene pairs. *ZoMYB#90* exhibited high expression levels in the rhizome bud, young flower bud, and root, whereas *ZoMYB#89* was not expressed in any organs or tissues. Notably, *ZoMYB#89* expression was significantly enhanced under ABA, cold, drought, heat, and salt treatments. Despite exposure to stress signals, *ZoMYB#90* exhibits minimal changes in response. In ZoMYB#90, the motifs Motif-3, Motif-1, and Motif-2 remain identical in composition and position, as depicted in Fig. 3. Consequently, we infer that the duplication of the *ZoMYB* gene has led to the emergence of novel functions and expression patterns. Furthermore, functional divergence may result in differential expression profiles of gene pairs. For instance, *ZoMAYB#3* exhibited high expression levels in the rhizome bud, while *ZoMYB#167* peaked in roots, as indicated in Supplementary Table S7. *ZoMYB#3* gene (motif-4, motif-3, motif-7, motif-1, motif-2, motif-8) exhibited the presence of motif 4 and motif 8, whereas *ZoMYB#167* (motif 3, motif 7, motif 1, motif 2) lacked these motifs (Fig. 3), suggesting that motif composition changes due to segmental duplication may contribute to functional divergence. Preliminary predictions of gene function can be made through analysis of gene expression patterns [57]. The results of the tissue expression patterns analysis revealed that a total of 36.49% (27/74) of 1R-MYB genes, 52.56% (82/156) of R2R3-MYB genes, and one 3R-MYB gene were expressed in all the tested tissues, as depicted in Fig. 7 and Supplementary Table S7. Notably, 16 *ZoMYBs* exhibited higher expression levels in roots compared to other tissues, which is consistent with findings in other plant species [58]. Furthermore, the flowering mechanism in ginger remains unclear. Our study reveals that *ZoMYB* genes, specifically 13, 21, and 24, are highly expressed in floral buds, young flowers, and mature flowers, respectively. Notably, *ZoMYB#22/63* are abundant in floral buds, while *ZoMYB#41/11/60/106* were more prevalent in young flowers, and *ZoMYB#73/84/149/122* were more abundant in mature flowers. It is noteworthy that genes that are high homologous in the same clade of a phylogenetic tree tend to have high sequence similarity and may perform similar functions [59, 60].

Based on phylogenetic analysis, *ZoMYB#92* exhibited significant homology with AtMYB80. In Arabidopsis, cotton, and Brassica, *MYB80* homologs have been revealed to play roles in the regulation of tapetal and pollen development, thereby providing a potential avenue for elucidating the function of *ZoMYB#92* [61]. In ginger, *ZoMYB#92* was exclusively expressed in mature flowers, suggesting it may play a role in regulating flower development. Further investigation is warranted to determine whether *ZoMYB#92* is also involved in the control of tapetal and pollen development in ginger. The flowering time in Arabidopsis is negatively affected by by MYR1 and MYR2 under reduced light intensity, as reported in [62]. In the case of ginger, the promoter of *ZoMYB#170*, which is the homolog of *MYR1* and *MYR2*, contains nine cis-elements that are responsive to light. This suggests that ZoMYB#170 may be involved in regulating the flowering time of ginger in response to light signals.

Given that rhizome expansion is a crucial factor affecting ginger production and thus has garnered significant attention in cultivation. Zhuang et al. (2022) have reported that in Arabidopsis, *MYB42* and *MYB85* are jointly responsible for the negative regulation of hypocotyl cell elongation [63]. Through phylogenetic analysis, it has been established that *ZoMYB#94/125* are orthologs of *MYB42/85*. *ZoMYB#94/125* were also highly expressed in the internodes of the rhizome, which is likely associated with rhizome elongation. Gingerols and curcuminoids are considered the most significant medicinal compounds in ginger. The rhizome of ginger contains a greater abundance of gingerols and curcuminoids compared to other organs. Li et al. (2021) reported that the expression patterns of seven MYB genes were similar to those of the enzyme genes involved in the biosynthesis of gingerols and curcuminoids [6]. Our study revealed that *ZoMYB#34/55/96/158/64/113* exhibited a gradual reduction from the young node (1st internode) to the old node (3rd internode) during rhizome development, while *ZoMYB#2/59/182/202* a gradual increased (Fig. 7). These findings suggest that these ZoMYBs may play roles in regulating the biosynthesis of gingerols and curcuminoids compounds. According to cis-element analysis, it was found that five R2R3-MYB genes (*ZoMYB#205/147/88/132/48*) and five MYB-related genes (*ZoMYB#24/124/207/117/65*) promoters contained flavonoid biosynthetic regulation element, indicating their regulatory function in flavonoid biosynthesis (Fig. 4).

The regulatory role of MYB TFs in plant responses to environmental stress has been extensively investigated [30, 51, 52]. As shown in Fig. 4 and Supplementary Table S2, most of the *ZoMYB* gene promoters contained hormone-related regulatory elements and abiotic stress-related regulatory elements, such as low temperature responsiveness, drought-inducibility, defense and stress responsiveness. These findings suggest *ZoMYB* genes may directly or indirectly modulate the response of ginger to abiotic stress. In general, environmental responsiveness elements, as as lower temperature, drought, and so on, were the most common in *ZoMYB* gene promoters. However, none of *ZoMYB* genes contained salt responsiveness element (Fig. 8 and Supplementary Table 8). These result suggest that the transcriptional regulation of different *ZoMYB* genes is diverse, indicating the multi-functionality of MYBs in ginger. In the present investigation, the expression patterns of selected *ZoMYB* genes exhibited significant fluctuations under various stress treatments, including ABA, cold, heat, drought, and salt. These findings suggest that these *ZoMYBs* may play key roles in ginger’s resistance to these stresses. Increasing evidence suggests that specific *MYB* genes are involved in the response to multiple stresses. For instance, *MYB49*-overexpressing tomato plants exhibited significant resistance to Phytophthora infestans, as well as tolerance to salt and drought stresses [64]. Similarly, in Arabidopsis, *MYB41* expression was not detected under normal growth conditions, but was found to be upregulated in response to high levels of drought, ABA, and salt stress [65]. Overexpression of *MYB41* in transgenic plants resulted in improved salinity tolerance during germination and root growth. In ginger, *ZoMYB#53/118* were also classified into this group.

*ZoMYB#53* and *ZoMYB#118* exhibited significant induction in response to salt, ABA, cold, and drought stress. Furthermore, *ZoMYB#118* also displayed induction in response to heat stress, while *ZoMYB#53* did not exhibit any response. The expression patterns of *ZoMYB#53* and *ZoMYB#118* differed in response to salt stress, with *ZoMYB#118* exhibiting a rapid response at 1 h, peaking at 12 h, and gradually decreasing within 24-48 h. In contrast, *ZoMYB#53* exhibited no response until 3 h after salt stress, and gradually increasing from 6 to 48 h, peaking at 48 h (Fig. 8b). Our analysis revealed that in ginger, the expression of *ZoMYB#11/36/105/204* gradually increased in response to salt from 1 to 48 hours, while *ZoMYB#60/202* gradually decreased. *AtMYB44* is activated under diverse abiotic stresses, including low temperature, dehydration, salinity, and ABA. Overexpression of *AtMYB44* lines resulted in a reduced rate of water loss and significantly improved tolerance to salt and drought stress compared to WT plants [34]. *ZoMYB#122/163/201* exhibited close homology with *AtMYB44*. In ginger, the expression of *ZoMYB#163* was significantly upregulated in response to cold and salt stress, while exhibiting negligible response to ABA, drought, and heat stress. Conversely, *ZoMYB#122* was markedly induced by all five stressors (ABA, cold, drought, heat, and salt), with cold stress instigating an amplification in expression exceeding 500-fold. On the other hand, *ZoMYB#201* displayed minimal variation in expression levels in response to these abiotic stresses. Overexpression of *AtMYB75* in transgenic lines led to a notable increase in flavonoid accumulation, which exhibited potent antioxidant activity and improved tolerance to abiotic stresses such as oxidative and drought stresses [66, 67]. In ginger, *ZoMYB#84*, *ZoMYB#193*, *ZoMYB#31*, and *ZoMYB#97* were grouped together with *AtMYB75*. *ZoMYB#84* exhibited a significant increase in expression levels in response to all five stressors except for salt stress, whereas *ZoMYB#31*, *ZoMYB#97*, and *ZoMYB#193* were only significantly induced by heat and salt stress. These patterns of gene expression intimate that the *ZoMYBs* may have a crucial function in responding to various stressors.

## Conclusions

This study conducted a methodical examination of the MYB family genes within *Z. officinale* Roscoe, culminating in the identification of 231 distinct MYB genes. These genes were unevenly distributed across 11 chromosomes. Phylogenetic analysis revealed the clustering of ZoMYBs into 37 subgroups, each characterized by congruent gene structures and motif compositions. Additionally, the synteny analysis demonstrated that *ZoMYB* genes had the highest number of orthologous gene pairs with MYB genes in *Musa acuminata*, followed by *Oryza sativa*. The findings of this study suggest that *ZoMYB* genes are integral to the developmental processes of ginger. Specifically, the gradual decrease in *ZoMYB#34/230/61/161/22/23/158/162* during rhizome developmental stages indicates their potential relevance in this biological process. Moreover, the analysis of RNA-Seq datasets revealed that 41 ZoMYB genes exhibited responses to abiotic stress. Notably, 39 *ZoMYBs* were induced by cold, 37 by drought and heat, 48 by ABA, and 62 by salt. In summary, these findings pave the way for further exploration of the functional roles of individual *MYB* genes in ginger.

### Supplementary Information

Supplementary Material 1.

Supplementary Material 2.

Supplementary Material 3.

Supplementary Material 4.

Supplementary Material 5.

Supplementary Material 6.

Supplementary Material 7.

Supplementary Material 8.

Supplementary Material 9.

Supplementary Material 10.

Supplementary Material 11.

Supplementary Material 12.

Supplementary Material 13.

Supplementary Material 14.

Supplementary Material 15.

Supplementary Material 16.

## Data Availability

The transcriptome data were deposited in the NCBI Short Read Archive (Project Accession Number: SRP064226).
