# Supplementary material for: The MYB family and their response to abiotic stress in ginger (Zingiber officinale Roscoe)
Source: BMC Genomics. 2024 May 11;25:460. doi: 10.1186/s12864-024-10392-1 (PMC11088133; doi:10.1186/s12864-024-10392-1)
Supplement: Supplementary file 10 — Supplementary Material 10. [file 12864_2024_10392_MOESM10_ESM.pdf]

1R,3R-MYB

		E-value	Site	Width
motif 1	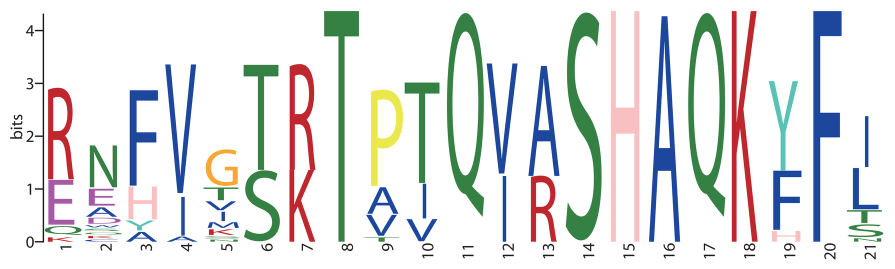	1.5 e-353	27	21
motif 2	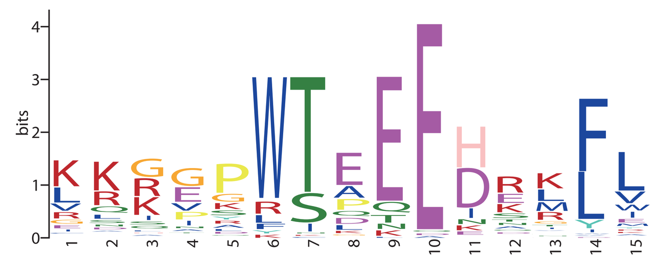	5.9 e-299	73	15
motif 3	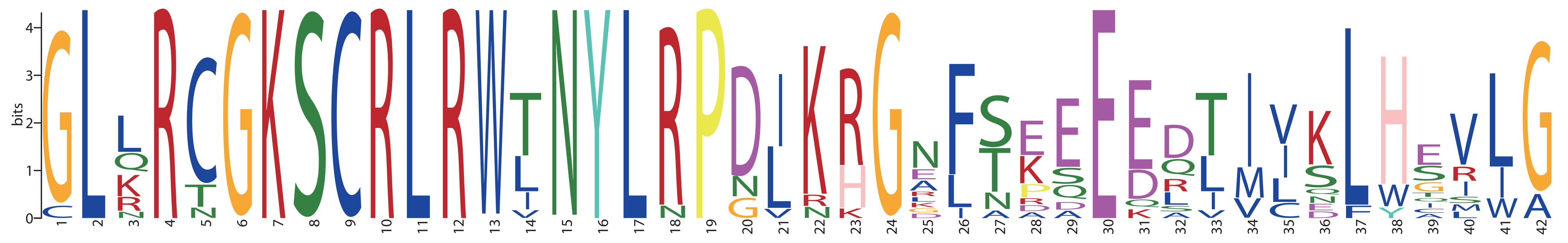	2.2 e-312	14	42
motif 4	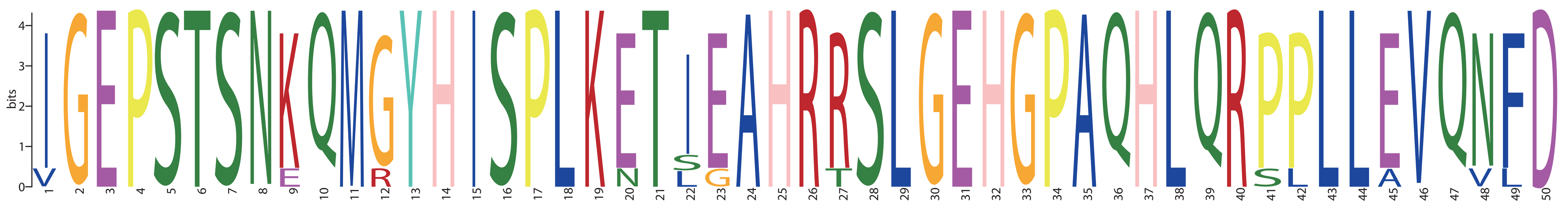	1.6 e-266	8	50
motif 5	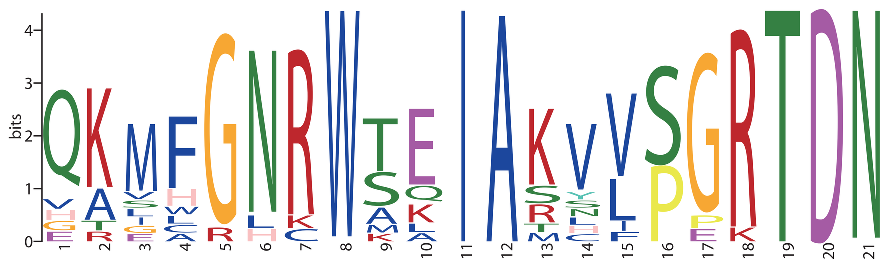	7.1 e-132	14	21
motif 6	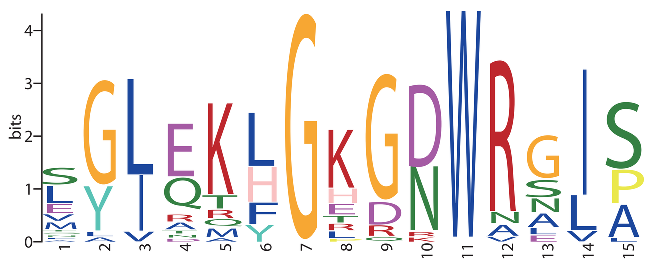	2.6 e-158	29	15
motif 7	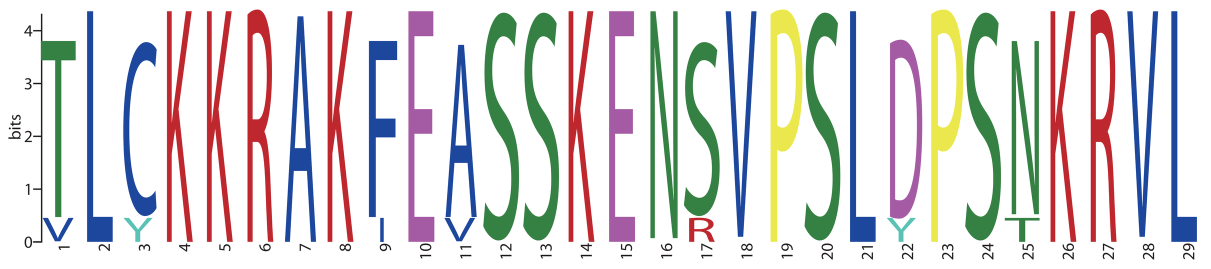	3.4 e-121	8	29
motif 8	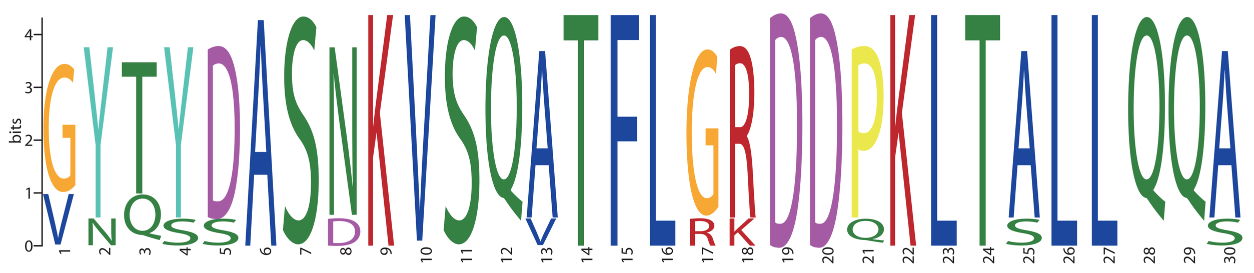	7.4 e-107	7	30
motif 9	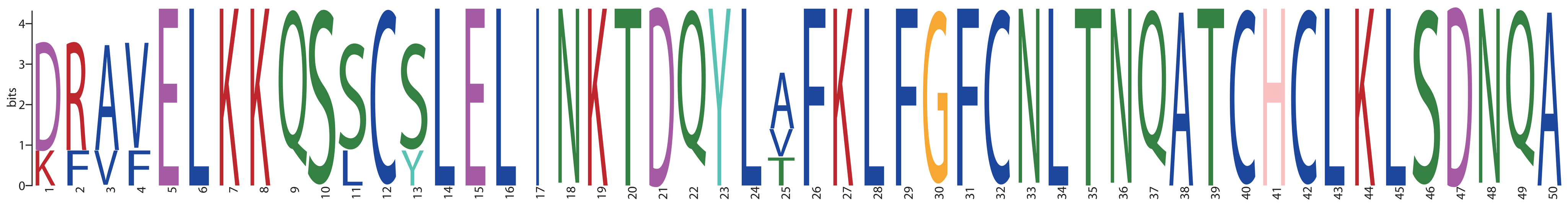	1.6 e-090	4	50
motif 10	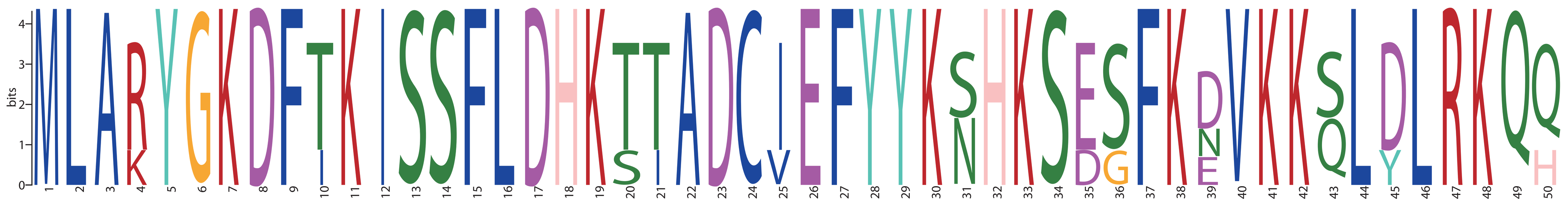	3.4 e-085	4	50

Supplementary Figure S1. Sequence logos for the conserved motifs of 1R-MYB and 3R-MYB ptoreins in ginger
