# Supplementary material for: The MYB family and their response to abiotic stress in ginger (Zingiber officinale Roscoe)
Source: BMC Genomics. 2024 May 11;25:460. doi: 10.1186/s12864-024-10392-1 (PMC11088133; doi:10.1186/s12864-024-10392-1)
Supplement: Supplementary file 11 — Supplementary Material 11. [file 12864_2024_10392_MOESM11_ESM.pdf]

# MYB-related

## R1 repeat

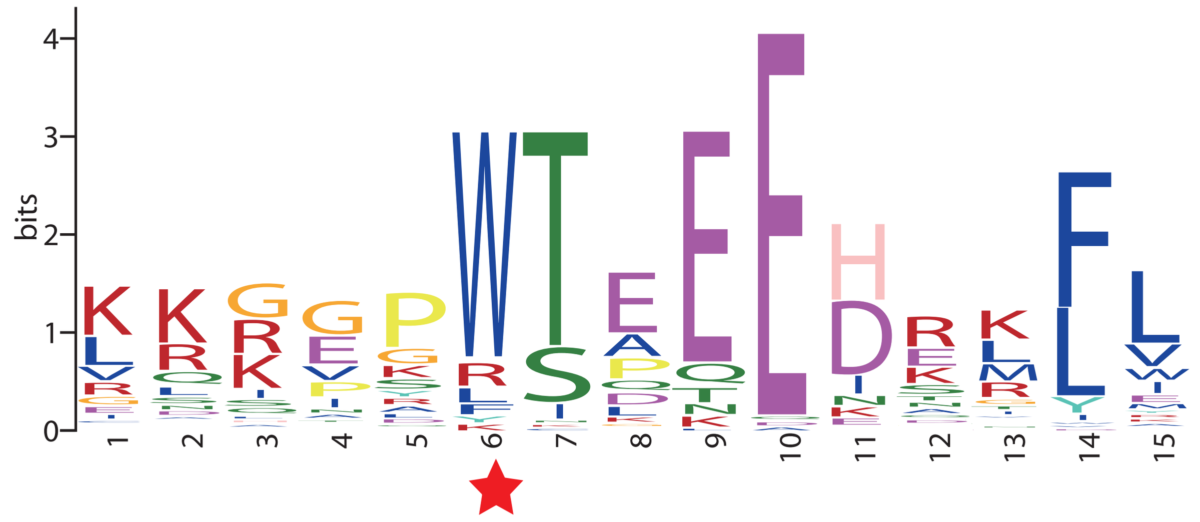

Motif 2

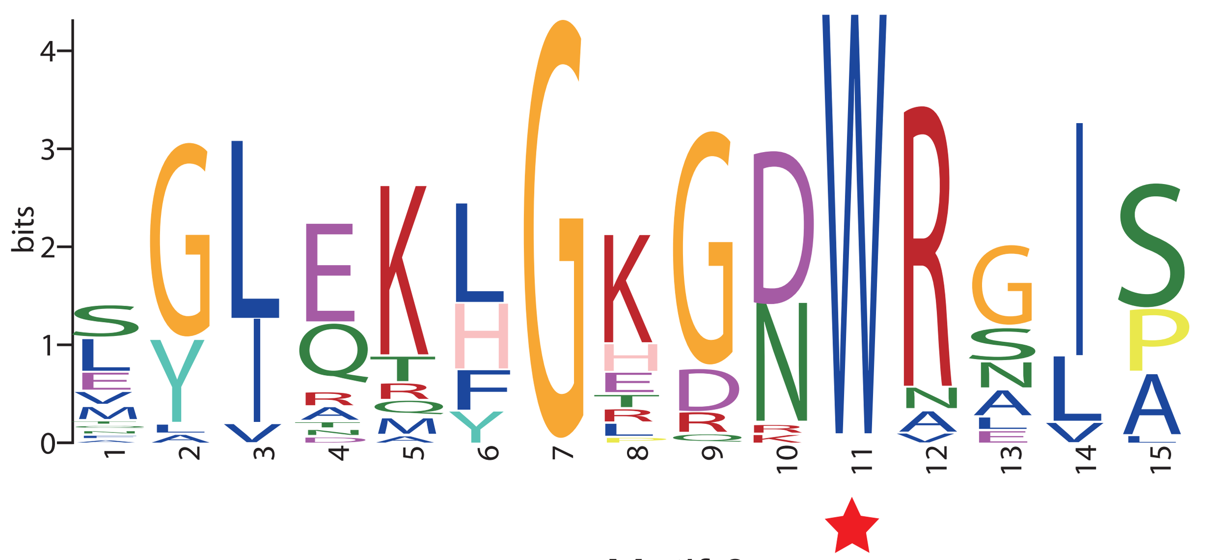

Motif 6

# R2R3-MYB

## R2 repeat

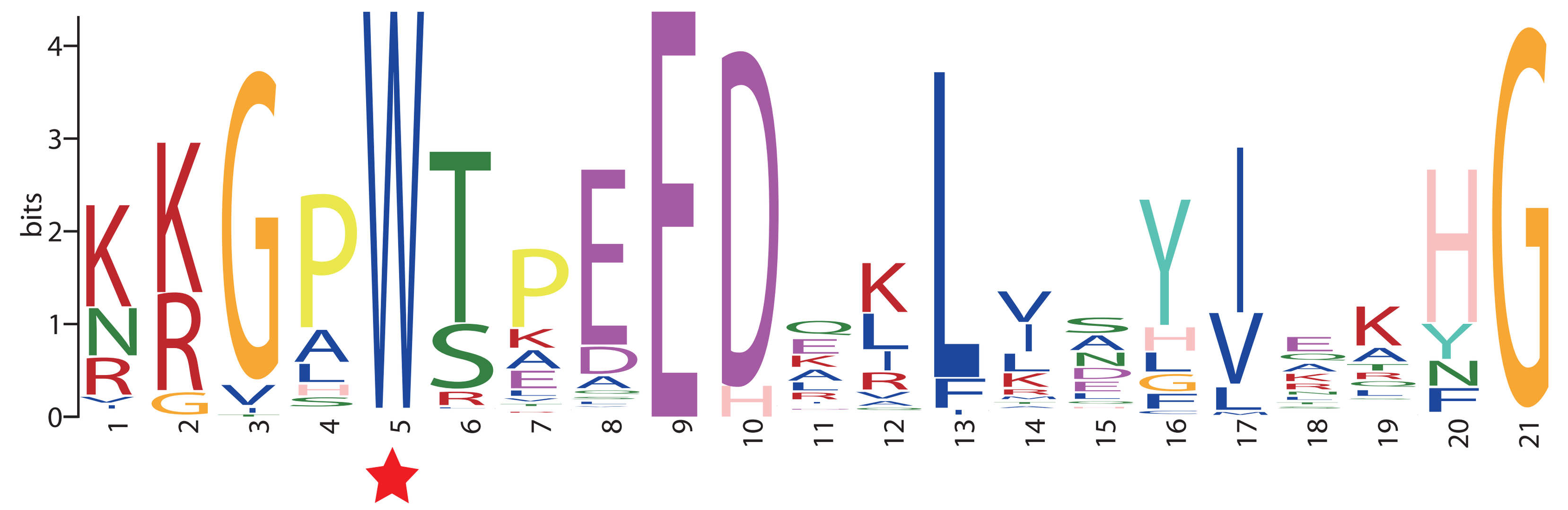

Motif 3

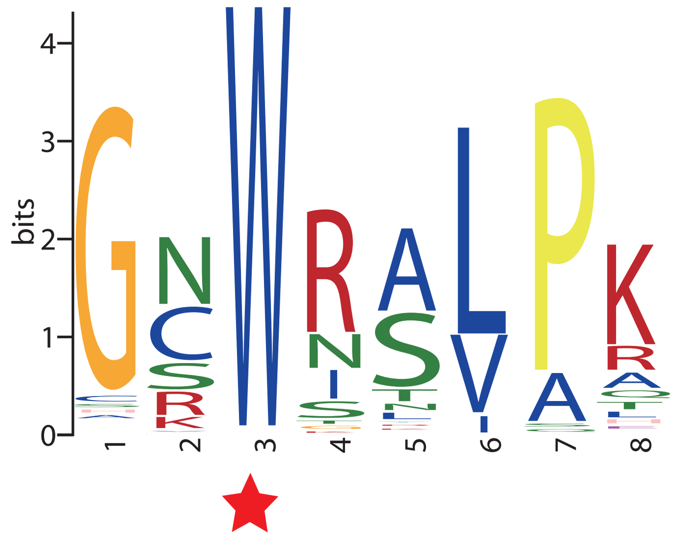

Motif 7

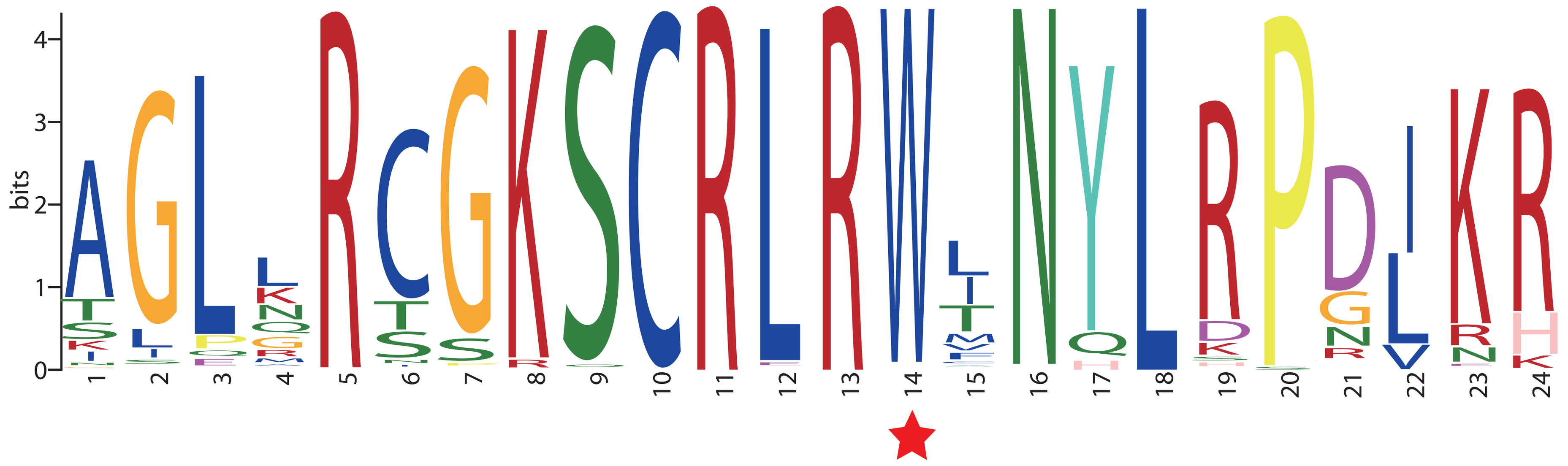

Motif 1

## R3 repeat

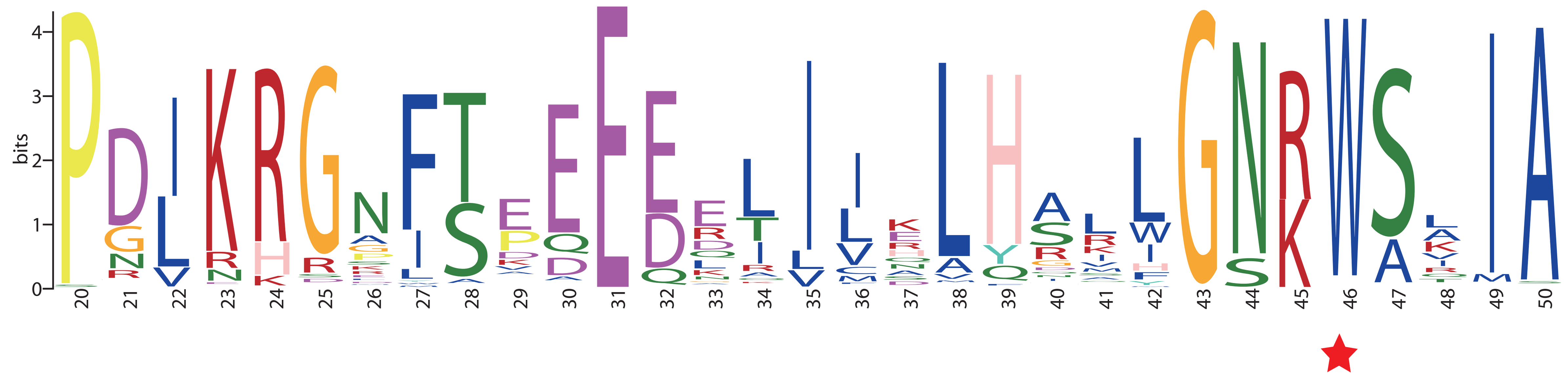

Motif 1 continued

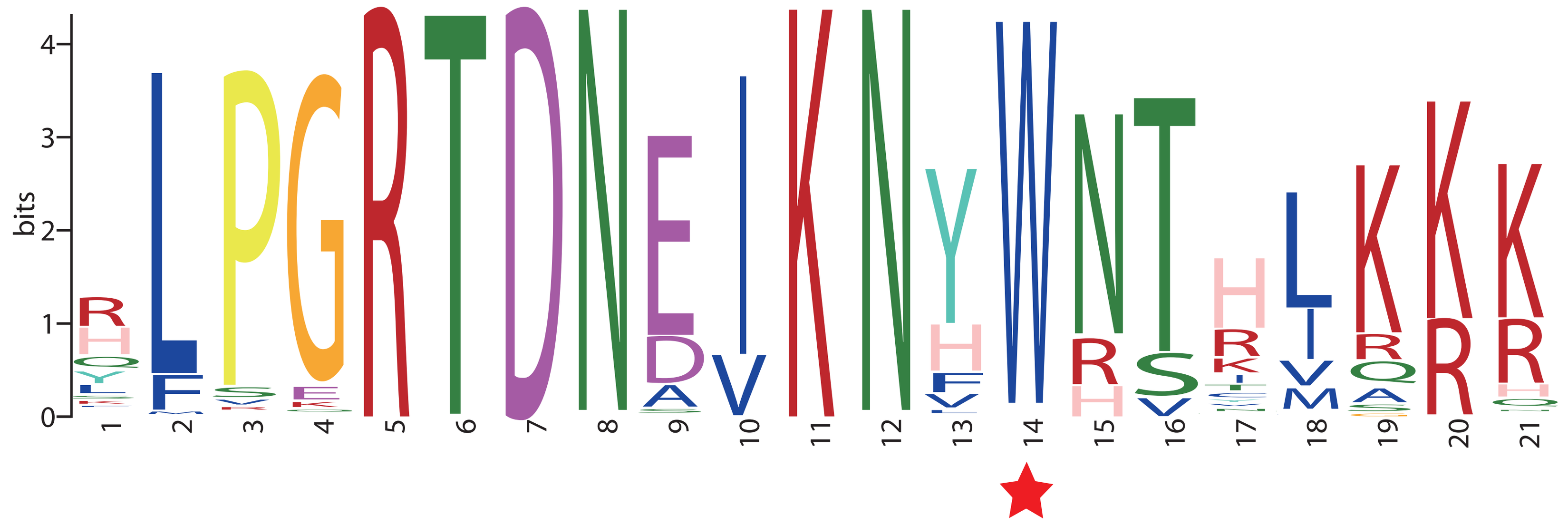

Motif 2

**Supplementary Figure S2.** R1, R2 and R3 MYB repeats of the proteins in ZoMYB gene family.
The overall height of each stack showed the conservation of the MYB protein sequence at that position. English letters indicate the different type of amino acid residue
