# Supplementary material for: The MYB family and their response to abiotic stress in ginger (Zingiber officinale Roscoe)
Source: BMC Genomics. 2024 May 11;25:460. doi: 10.1186/s12864-024-10392-1 (PMC11088133; doi:10.1186/s12864-024-10392-1)
Supplement: Supplementary file 12 — Supplementary Material 12. [file 12864_2024_10392_MOESM12_ESM.pdf]

R2R3-MYB

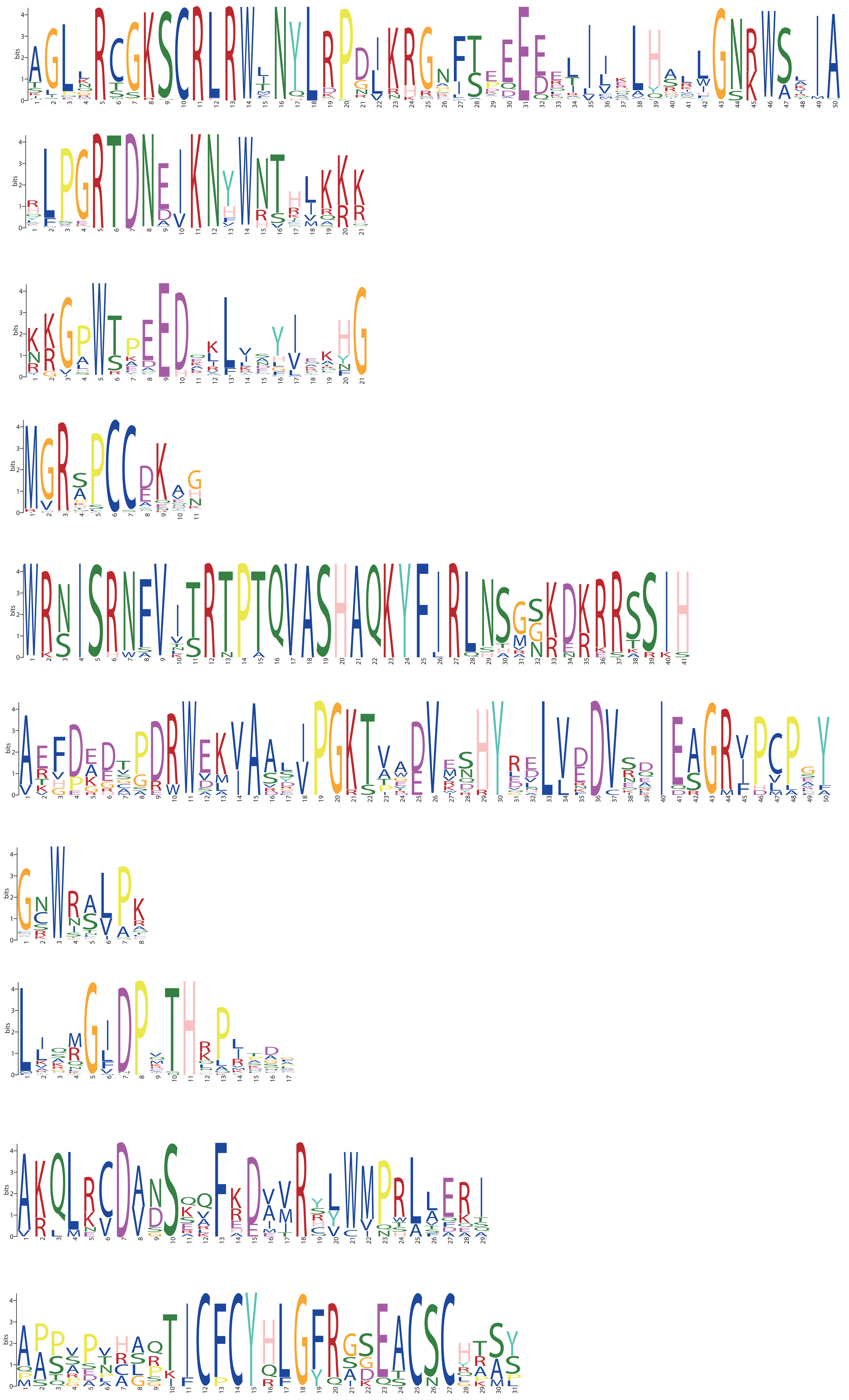

Motif	E-value	Site	Width
motif 1	6.5 e-5159	137	50
motif 2	2.3 e-2355	140	21
motif 3	1.3 e-1740	154	21
motif 4	6.0 e-545	85	11
motif 5	1.4 e-450	15	41
motif 6	2.7 e-365	15	50
motif 7	2.4 e-339	118	8
motif 8	8.9 e-255	44	17
motif 9	3.6 e-131	12	29
motif 10	2.3 e-099	9	31

Supplementary Figure S3. Sequence logos for the conserved motifs of R2R3-MYB ptoreins in ginger
