# Supplementary material for: The MYB family and their response to abiotic stress in ginger (Zingiber officinale Roscoe)
Source: BMC Genomics. 2024 May 11;25:460. doi: 10.1186/s12864-024-10392-1 (PMC11088133; doi:10.1186/s12864-024-10392-1)
Supplement: Supplementary file 14 — Supplementary Material 14. [file 12864_2024_10392_MOESM14_ESM.pdf]

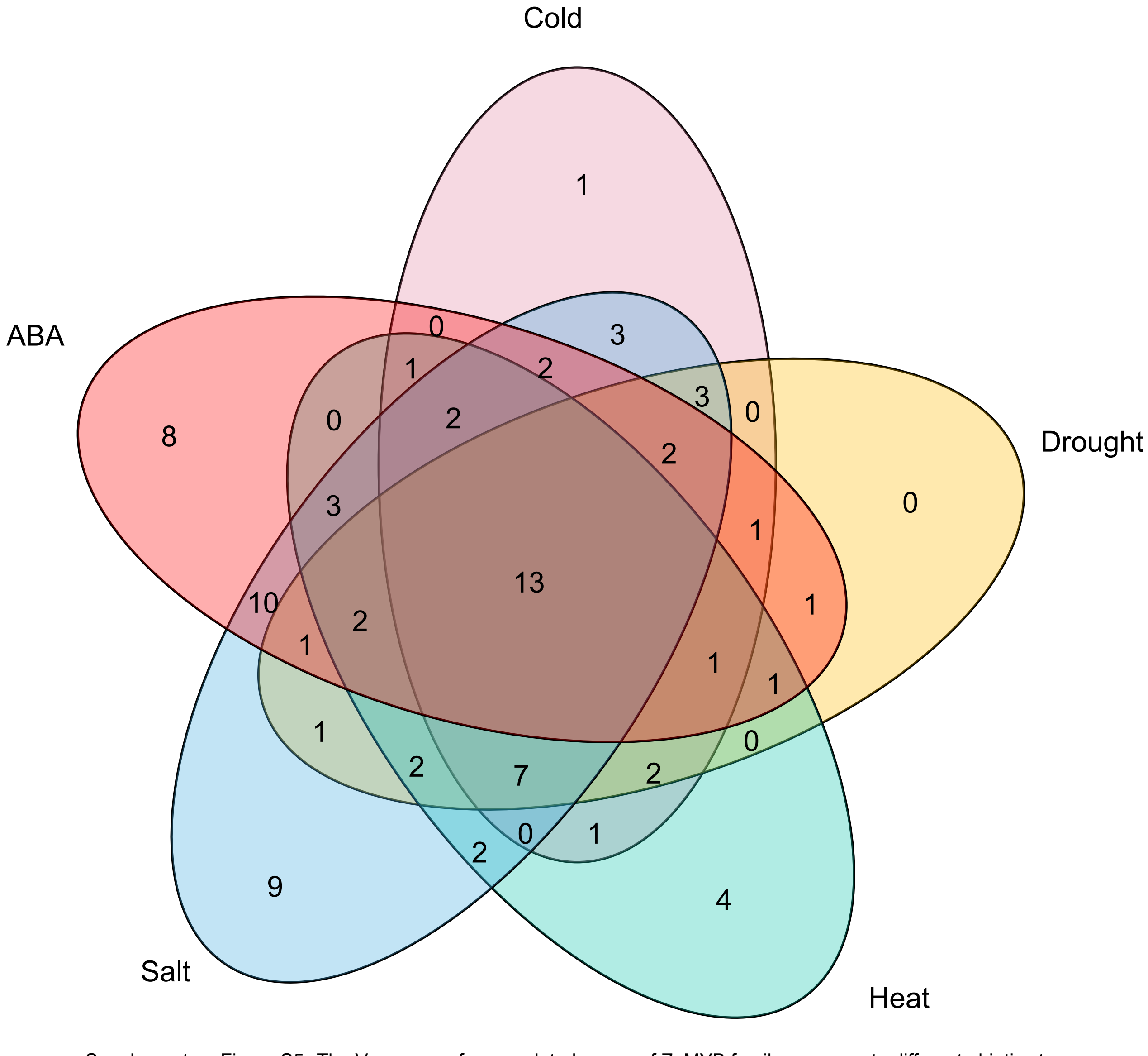

Supplementary Figure S5. The Venn map of up regulated genes of ZoMYB family response to different abiotic stresses
