# Supplementary material for: The MYB family and their response to abiotic stress in ginger (Zingiber officinale Roscoe)
Source: BMC Genomics. 2024 May 11;25:460. doi: 10.1186/s12864-024-10392-1 (PMC11088133; doi:10.1186/s12864-024-10392-1)
Supplement: Supplementary file 15 — Supplementary Material 15. [file 12864_2024_10392_MOESM15_ESM.pdf]

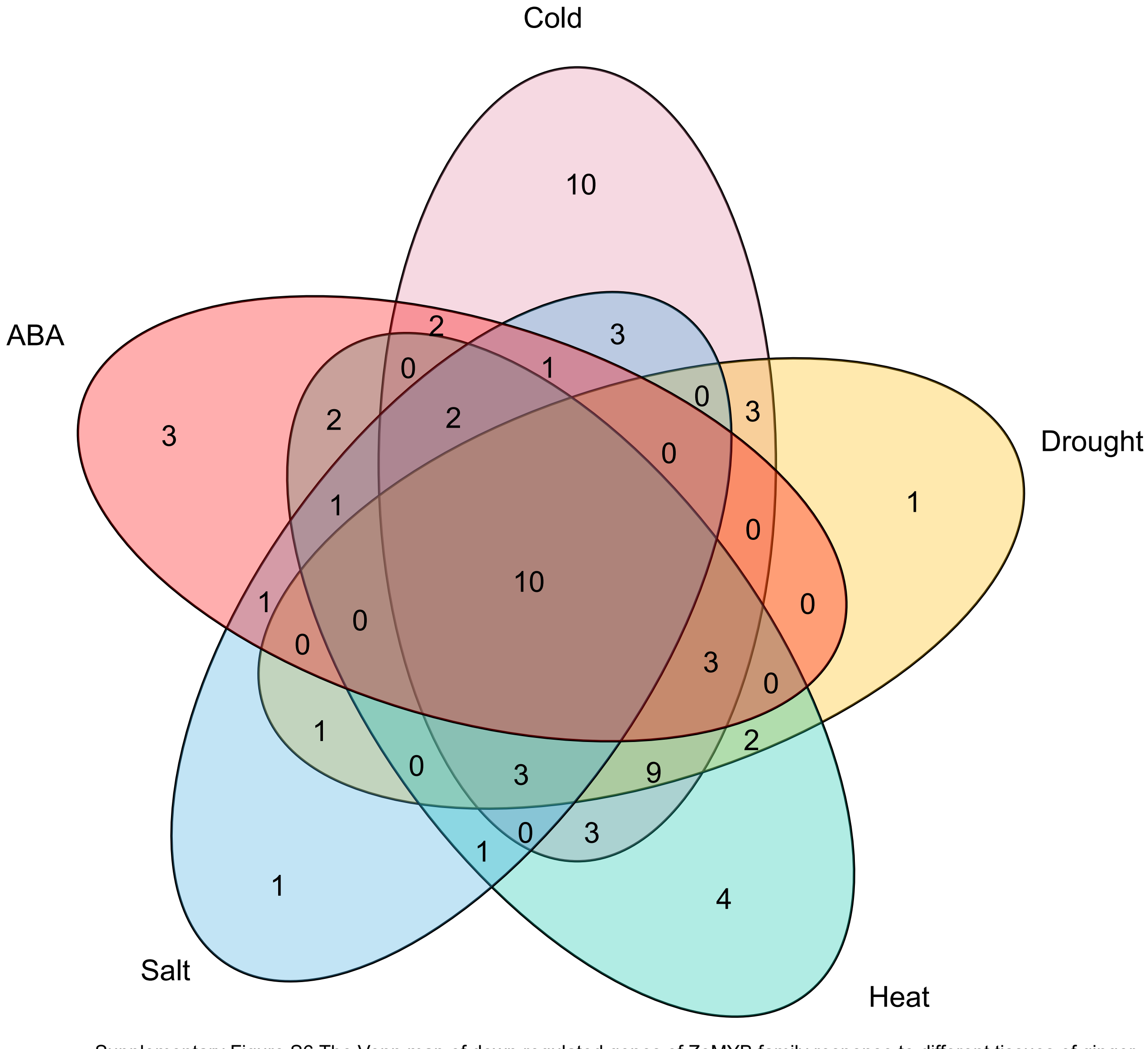

Supplementary Figure S6.The Venn map of down regulated genes of ZoMYB family response to different tissues of ginger
