# Supplementary material for: The MYB family and their response to abiotic stress in ginger (Zingiber officinale Roscoe)
Source: BMC Genomics. 2024 May 11;25:460. doi: 10.1186/s12864-024-10392-1 (PMC11088133; doi:10.1186/s12864-024-10392-1)
Supplement: Supplementary file 16 — Supplementary Material 16. [file 12864_2024_10392_MOESM16_ESM.pdf]

Supplemetary Document S1. A phylogram of ginger and Arabidopsis MYBs in newick format.

((('AT1G18710.1':0.4992600000000005,'AT1G74430.1':0.30389)1.0:0.9394800000000005,((('AT
5G07690.1':0.16597000000000062,'AT5G07700.1':0.2561600000000004)0.926:0.191729999999
99973,'AT5G61420.1':0.34450000000000003)1.0:0.7362900000000003,('AT5G60890.1':0.90598
00000000005,('AT1G18570.1':0.22818999999999967,'AT1G74080.1':0.3410000000000002)1.0:0.
6217000000000006)0.86:0.1997)0.951:0.24085)0.79:0.09619999999999962,(((((((((((('ZoMYB#
176':0.23399999999999999,'ZoMYB#106':0.11954999999999938)0.995:0.3789699999999998,('
ZoMYB#86':0.2671000000000001,'ZoMYB#187':0.30161000000000016)0.059:0.150590000000
00022)0.997:0.3913600000000006,('AT1G25340.1':0.5817300000000003,'AT1G68320.1':0.4596
900000000015)0.991:0.34281000000000006)0.968:0.23005999999999993,((('ZoMYB#36':0.27
54999999999992,'ZoMYB#144':0.40675999999999934)0.997:0.43106000000000044,(('ZoMYB
#191':0.13008000000000042,'ZoMYB#82':2.53109)0.909:0.35924000000000067,('AT3G01530.1':
0.6439599999999999,('AT3G27810.1':0.19406000000000034,'AT5G40350.1':0.225719999999999
992)0.96:0.19561999999999999)0.57:0.019090000000000273)0.994:0.47743999999999964)0.929:
0.20941999999999972,((((('ZoMYB#174':0.06235999999999997,'ZoMYB#105':0.243570000000
00006)0.986:0.22860000000000014,('ZoMYB#11':0.6351299999999993,('ZoMYB#204':0.12931
999999999988,'ZoMYB#57':0.13153999999999932)0.944:0.18890999999999999)0.327:0.114230
00000000094)0.913:0.13915999999999995,'ZoMYB#179':0.6214900000000005)0.394:0.0580799
99999999465,('ZoMYB#220':0.26900000000000013,'ZoMYB#212':0.6129699999999998)0.99:0.
32845999999999975)0.959:0.19108000000000036,(('AT1G48000.1':0.7398699999999998,('AT3
G06490.1':0.22160000000000046,'AT5G49620.1':0.18843999999999994)0.973:0.293239999999
99906)0.62:0.11667000000000094,'AT2G47190.1':0.8807100000000005)0.79:0.07778999999999
936)0.952:0.19780999999999996)0.909:0.14037000000000077)0.976:0.35183999999999926,(('A
T3G30210.1':0.8654599999999997,((('ZoMYB#181':0.5134699999999999,'ZoMYB#167':1.0986
799999999999)0.453:0.11125000000000007,'ZoMYB#152':0.5716799999999997)0.933:0.27670
999999999957,('AT3G24310.1':0.08983000000000008,'AT4G13480.1':0.31650000000000045)0.
998:0.5681999999999992)0.198:0.12748999999999988)0.997:0.7615100000000004,('AT3G5320
0.1':1.1741000000000001,('ZoMYB#77':1.15883,(('AT3G46130.1':0.13230000000000075,'AT5G
59780.1':0.10707000000000022)0.981:0.41641999999999957,('ZoMYB#231':1.05890999999999
91,(('ZoMYB#214':0.1577200000000003,'ZoMYB#134':0.25933999999999999)0.873:0.18944000
000000027,('ZoMYB#205':0.08237000000000005,'ZoMYB#56':0.20162000000000013)1.0:0.484
23000000000016)0.829:0.2227699999999997)0.869:0.26456000000000035)0.878:0.2951699999
999997)0.924:0.4268100000000006)0.811:0.30616999999999983)0.758:0.11513999999999935)
0.328:0.3596400000000006,'AT5G47290.1':7.6157299999999999)0.405:0.2572000000000001,'Zo
MYB#180':2.33193)0.247:0.08448000000000011,(((((((('ZoMYB#89':0.37699000000000016,'Zo
MYB#100':0.42391999999999985)0.992:0.5118299999999998,(('AT1G26780.1':0.49498999999
99996,'AT1G69560.1':0.57437)0.959:0.3559599999999996,('AT3G29020.1':0.694200000000000
4,'AT5G39700.1':1.5695699999999997)0.936:0.47733000000000025)0.947:0.363229999999999
7)0.795:0.2753300000000003,('AT5G17800.1':0.9609199999999998,('AT1G17950.1':0.37859999
99999996,'AT1G73410.1':0.20345000000000013)0.997:0.5993599999999999)0.567:0.14965999
99999999)0.303:0.08535000000000004,('AT4G33450.1':1.1378199999999996,('ZoMYB#213':0.
7321600000000004,('ZoMYB#215':0.6783299999999999,'ZoMYB#98':0.3276399999999997)0.8
68:0.20221)0.812:0.25900999999999996)0.874:0.17513000000000023)0.824:0.09508999999999
99,('ZoMYB#95':0.8866699999999996,(('ZoMYB#133':0.4209699999999996,('ZoMYB#18':0.69
70900000000002,'ZoMYB#48':0.3366800000000003)0.147:0.12526999999999955)0.862:0.1869

8000000000015,'ZoMYB#112':0.9801000000000002)0.655:0.1537600000000012)0.103:0.1245
9000000000042)1.0:0.9929899999999998,(('ZoMYB#201':0.9978499999999997,(('ZoMYB#163':
0.20479999999999965,'ZoMYB#122':0.08704)0.982:0.3140200000000002,(('AT2G23290.1':0.20
73999999999998,'AT4G37260.1':0.15566000000000013)0.997:0.26365000000000016,('AT3G50
060.1':0.3399700000000001,'AT5G67300.1':0.20950000000000024)0.984:0.2217500000000001)
0.986:0.30825000000000014)0.826:0.16948000000000008)0.989:0.5368099999999991,(('AT2G3
9880.1':0.4564899999999996,'AT3G55730.1':0.2607600000000003)0.996:0.4648300000000001,(
'ZoMYB#230':0.45640000000000036,'AT3G09230.1':0.78803)0.837:0.25283000000000033)0.99
8:0.68295)0.963:0.3696200000000003)0.977:0.3587600000000002,((('AT5G11510.1':0.52948000
00000004,('AT4G32730.1':0.44516000000000044,'ZoMYB#131':0.7406800000000002)0.502:0.1
9017000000000017)1.0:0.6782599999999999,(('AT3G09370.1':0.30098999999999965,('AT2G13
960.1':0.14733000000000018,'AT5G02320.1':0.000810)1.0:0.24429999999999996)0.994:0.37988
99999999996,('ZoMYB#226':0.6338299999999997,'ZoMYB#115':0.47050000000000036)0.997:
0.4072399999999998)0.847:0.21344999999999992)1.0:0.9281999999999995,((('AT5G11050.1':0.
2667799999999998,'AT5G58850.1':0.13673999999999964)1.0:0.9296199999999999,('AT4G187
70.1':1.0137100000000006,('AT3G27785.1':0.4536400000000004,(('AT2G25230.1':0.16856000
000000027,'AT5G40430.1':0.16758000000000006)1.0:1.1052999999999997,'AT5G40360.1':0.30
586999999999964)0.98:0.4015599999999999)0.995:0.5281900000000004)0.435:0.21389000000
000014)1.0:1.0805300000000004,('AT1G43330.1':4.33678,'AT4G00540.1':0.5955000000000004)
0.568:0.8091200000000001)0.306:0.057469999999999466)0.923:0.22031000000000045)0.942:0.
5394199999999998,(((('AT1G14350.1':0.10714999999999986,'AT2G02820.1':0.21372999999999
998)0.992:0.6336199999999996,('ZoMYB#129':0.3470500000000003,(((('ZoMYB#117':0.01438
000000000006,('ZoMYB#186':0.025470000000000326,'ZoMYB#65':0.033489999999999576)0.5
26:0.000820)0.0:0.000830,'ZoMYB#199':0.009949999999999903)0.755:0.000840,'ZoMYB#173':
0.00527000000000033)0.785:0.000820,('ZoMYB#103':0.09973999999999972,'ZoMYB#15':0.20
544999999999999)0.681:0.0389400000000002)1.0:0.42828)0.997:0.6892100000000001)1.0:1.043
87,((((('AT1G26580.1':3.5109499999999993,((((('AT5G59570.1':1.0706699999999998,('AT2G40
970.1':0.3717699999999997,('AT3G10760.1':0.1277999999999997,'AT5G05090.1':0.2749300000
0000034)0.897:0.29330000000000034)0.863:0.30672999999999995)0.993:1.1838100000000003,
('AT1G49560.1':1.25704,('AT4G37180.1':1.3663699999999999,('AT2G03500.1':0.976990000000
0007,(('AT1G13300.1':0.2661799999999994,'AT3G25790.1':0.4231699999999998)0.979:0.27653
99999999998,('AT1G25550.1':0.41780999999999935,'AT1G68670.1':0.29726999999999926)0.9
74:0.2694200000000002)0.998:0.47902999999999984)0.833:0.23250999999999955)0.86:0.3488
9999999999954)0.998:1.0884999999999998)0.819:0.59274,(((('AT1G69580.1':0.7713600000000
005,(('AT3G04030.1':0.10587999999999997,'AT5G18240.1':0.11587999999999976)0.999:0.5557
2,('AT3G12730.1':0.7186899999999996,'AT3G24120.1':0.44686999999999966)0.991:0.4384800
000000002)0.784:0.18721999999999994)0.998:0.48367999999999967,('AT2G01060.1':0.505130
0000000003,'AT5G29000.1':0.7905800000000003)0.01:0.18794999999999984)0.692:0.0983299
999999998,'AT2G20400.1':1.1760200000000003)0.811:0.09905000000000008,('AT3G13040.1':1.
2040199999999999,'AT5G06800.1':0.9097)0.726:0.10252999999999979)1.0:1.40536)0.852:0.50
47199999999998,('AT4G04580.1':1.6955900000000002,('AT2G06020.1':1.6640100000000002,('
AT2G40260.1':0.8463000000000003,'AT2G42660.1':0.9951100000000004)0.847:0.43196000000
00001)0.836:0.3728800000000003)0.882:0.4074)0.996:0.9343500000000002,((('ZoMYB#208':0.
23920999999999992,'ZoMYB#96':0.13773000000000035)0.984:0.19632999999999967,('ZoMY

B#229':0.23620999999999998,'ZoMYB#49':0.24010999999999996)0.953:0.17218999999999962)1.
0:1.96833,(('ZoMYB#114':0.8484499999999997,((('ZoMYB#32':0.7664499999999999,'ZoMYB#
222':0.34856)0.985:0.44428999999999963,((('AT3G10113.1':0.6697300000000004,('ZoMYB#22
7':0.5636300000000007,('ZoMYB#2':0.4005000000000001,'ZoMYB#40':0.37089999999999998)0.
855:0.18505000000000038)0.981:0.31602999999999996)0.995:0.35287999999999986,'ZoMYB#
182':0.7440899999999999)0.801:0.2312000000000003,'AT5G17300.1':0.90109)0.518:0.19977)1.
0:0.9855299999999998,('AT3G09600.1':0.4860800000000003,(('ZoMYB#37':0.44930000000000
003,('ZoMYB#33':0.10369000000000028,'ZoMYB#61':0.15315000000000012)0.995:0.20551999
999999992)0.772:0.08495999999999997,('AT5G52660.1':0.40977999999999996,('AT1G01520.1':0.
2968000000000002,'AT4G01280.1':0.25753999999999966)1.0:0.40468999999999955)0.966:0.1
6915000000000013)0.779:0.11746000000000034)0.986:0.50115)0.865:0.2780300000000002)1.0:
1.2555399999999999,(((('AT1G19000.1':0.34013000000000027,'AT1G74840.1':0.327759999999
9996)1.0:1.0137099999999997,((('ZoMYB#159':0.04511999999999983,'ZoMYB#210':0.260779
99999999957)0.999:0.26525,('ZoMYB#63':0.18675999999999996,'ZoMYB#30':0.218980000000
0017)0.997:0.23629000000000033)0.969:0.21506000000000025,('AT3G16350.1':0.5544000000
00002,('AT5G47390.1':0.28406999999999998,('ZoMYB#55':0.1740700000000004,('ZoMYB#135':
0.049140000000000406,'ZoMYB#216':0.08035000000000014)0.994:0.1492300000000002)0.991:
0.20223000000000013)1.0:0.36470999999999965)0.274:0.07843)0.815:0.15658999999999956)0.
72:0.27324000000000037,('AT5G61620.1':1.2808799999999998,('ZoMYB#123':1.07723999999
99998,('ZoMYB#155':0.24424000000000046,('ZoMYB#207':0.89337,('ZoMYB#166':0.1745700
0000000011,'ZoMYB#188':0.22958999999999996)0.85:0.10299999999999976)0.78:0.09525000
000000006)0.967:0.40991999999999996)0.892:0.32336999999999997)0.612:0.0711500000000002
7)1.0:1.14386,(((((('ZoMYB#50':0.5768300000000002,('ZoMYB#62':0.48008000000000006,('Zo
MYB#128':0.23446000000000033,('ZoMYB#228':0.20230999999999977,('ZoMYB#203':0.0764
1999999999971,'ZoMYB#59':0.20092)0.972:0.08499000000000034)0.805:0.0538800000000003
7)0.945:0.13086999999999982)0.737:0.10806000000000004)0.704:0.08450000000000024,(('Zo
MYB#169':0.29938999999999998,'ZoMYB#46':0.22459999999999997)1.0:0.45467000000000013,
('ZoMYB#143':0.3752500000000003,('ZoMYB#124':0.12497999999999987,'ZoMYB#93':0.1586
2999999999996)0.966:0.16429999999999999)0.982:0.20394000000000023)0.382:0.098250000000
00017)0.991:0.3152600000000003,(('AT3G11280.1':0.16166999999999998,'AT5G05790.1':0.234
9800000000002)1.0:0.8061800000000003,('AT5G58900.1':0.5321400000000001,('AT2G38090.1':
0.22334999999999994,'AT5G01200.1':0.6387299999999998)0.972:0.2706200000000001)0.957:
0.28354000000000035)0.884:0.1419100000000002)0.957:0.3531500000000003,('AT4G36570.1':
0.15078000000000014,('ZoMYB#171':0.5787100000000001,('ZoMYB#175':0.330480000000000
55,('AT1G19510.1':0.3510900000000001,'AT1G75250.1':0.39126999999999956)0.759:0.165359
99999999973)0.94:0.50021)0.145:0.12988999999999962)0.998:1.2185699999999997)0.842:0.16
018000000000043,((('AT1G49010.1':0.6152100000000003,('ZoMYB#6':0.29262999999999995,('
ZoMYB#23':0.09567999999999977,('ZoMYB#164':0.046820000000000306,'ZoMYB#110':0.301
89999999999984)0.979:0.12094000000000005)0.993:0.24307000000000034)0.999:0.40008)0.95
6:0.21980999999999984,(('ZoMYB#223':0.9424299999999999,('ZoMYB#161':0.183019999999
99996,'ZoMYB#120':0.11235999999999998)0.883:0.08858999999999995)0.766:0.118319999999
99976,'AT5G08520.1':0.33732999999999997)0.632:0.14724999999999966)0.965:0.26837999999
99996,'AT5G23650.1':1.1665799999999997)0.893:0.2408400000000004)0.883:0.203999999999
99974,(('AT3G10590.1':1.21291,('AT3G10585.1':0.5922799999999997,('AT3G10580.1':0.339039

9999999998,'AT4G09450.1':0.38734999999999964)0.809:0.1783999999999999)0.928:0.338930
0000000004)0.992:0.6039700000000003,(('AT3G10595.1':2.3554200000000005,'AT5G04760.1':
0.55715)0.208:0.2324200000000003,('ZoMYB#111':0.3650700000000002,('ZoMYB#109':0.1270
6000000000017,'ZoMYB#24':0.18689)0.97:0.3589000000000002)0.999:0.8607899999999997)0.
223:0.22144999999999992)0.612:0.02958000000000016)0.88:0.3943399999999997)1.0:1.38478
00000000001)0.768:0.2706400000000002)0.456:0.09039999999999981)0.575:0.1051700000000
0021)0.727:0.19539999999999935,'ZoMYB#225':3.7400799999999999)0.623:0.01592000000000
038,(('AT2G47210.1':2.07475,('ZoMYB#28':0.12570999999999977,'ZoMYB#137':0.0698100000
0000037)1.0:2.01464)0.79:0.39534999999999965,('AT5G45420.1':2.9203199999999994,'AT5G4
1020.1':2.2618400000000003)0.873:0.5218099999999994)0.911:0.3720600000000003)0.899:0.2
274699999999994,'AT2G37630.1':2.4204599999999994)0.343:0.19753000000000043,('AT1G35
516.1':5.18317,('AT1G09770.1':2.03805,('AT3G18100.1':0.7333300000000005,'ZoMYB#197':0.6
475200000000001)1.0:1.3619700000000003)0.554:0.3764599999999998)0.426:0.000820)0.009:
0.18653999999999993)0.566:0.18424999999999958,('ZoMYB#38':1.62256,('AT1G58220.1':0.60
20300000000001,('ZoMYB#157':0.66134,('ZoMYB#170':0.18391000000000002,'ZoMYB#47':0.
13727)0.897:0.3444200000000004)1.0:1.5196199999999997)0.989:1.3233299999999995)0.992:
0.9781199999999997)0.616:0.14101000000000052)0.999:0.55131)0.617:0.1372800000000005,((
'ZoMYB#132':1.1509900000000002,(((('AT3G11440.1':0.29720000000000013,'AT5G06100.1':0.
27784999999999993)0.999:0.48965000000000014,('ZoMYB#81':0.24882000000000026,'ZoMY
B#154':0.30382)1.0:0.49366999999999983)0.999:0.5261100000000001,('AT4G26930.1':0.31198
000000000015,'AT5G55020.1':0.25289999999999946)1.0:0.7437200000000006)0.949:0.254560
00000000056,'AT2G32460.1':0.8427900000000008)0.12:0.03509999999999991)0.947:0.330769
99999999934,('AT2G26950.1':0.67272,'AT2G26960.1':0.25176)1.0:1.0269199999999996)0.989:0.
37418000000000085)0.962:0.2425499999999996,(((('ZoMYB#69':0.7785400000000005,'AT1G7
1030.1':1.1417900000000003)0.585:0.1513,((('ZoMYB#29':0.6407799999999995,'ZoMYB#162':
0.5545499999999999)0.883:0.1796799999999994,(('ZoMYB#34':0.7191400000000003,('ZoMY
B#5':0.3784900000000002,((('ZoMYB#202':0.24233999999999956,'ZoMYB#60':0.10311999999
999966)0.902:0.1216999999999997,('ZoMYB#149':0.09454999999999991,'ZoMYB#41':0.19948
000000000032)0.958:0.15065999999999935)0.959:0.2298100000000005,(('ZoMYB#3':0.095230
00000000081,'ZoMYB#16':0.09432000000000063)0.731:0.11221999999999976,('ZoMYB#119':
0.18400999999999978,'ZoMYB#160':0.17870000000000008)0.98:0.1849400000000001)0.997:0.
3136000000000001)0.607:0.11674000000000007)0.887:0.15929000000000038)0.076:0.0884199
9999999928,('AT1G22640.1':0.6392900000000008,('AT1G35515.1':0.39000000000000057,'AT4
G09460.1':0.13803000000000054)0.945:0.2524899999999999)0.982:0.3436799999999991)0.716:
0.15216999999999992)0.669:0.09999000000000002,(((('ZoMYB#102':0.3992899999999997,('Z
oMYB#177':0.22125999999999912,'ZoMYB#221':0.4337799999999996)0.876:0.180559999999
99983)0.813:0.16813000000000056,('ZoMYB#209':0.3396099999999995,'ZoMYB#158':0.36677
999999999944)0.272:0.11258000000000035)0.58:0.15160000000000018,('AT4G38620.1':0.3776
5999999999966,('AT2G16720.1':0.2558499999999997,'AT4G34990.1':0.31845999999999997)0.
996:0.30044000000000004)0.859:0.2030000000000003)0.936:0.22475000000000023,'ZoMYB#
150':0.44522999999999957)0.356:0.04225999999999974)0.996:0.46372000000000035)0.948:0.3
043099999999992,((('ZoMYB#79':0.014590000000000103,'ZoMYB#78':0.01717000000000013)
0.994:0.5558100000000001,('AT5G49330.1':0.7731500000000002,('AT2G47460.1':0.228040000
00000002,'AT3G62610.1':0.3967200000000002)0.994:0.43717000000000006)0.962:0.38818000

00000002)0.795:0.44309000000000065,(('ZoMYB#193':0.7663000000000002,'ZoMYB#97':0.44
820999999999955)0.851:1.0979700000000001,('ZoMYB#84':1.4931200000000002,('AT2G3042
0.1':0.84138,'ZoMYB#31':1.5592699999999997)0.824:0.32423)0.948:1.1804300000000003)0.99
9:2.8185400000000005)0.765:0.21934999999999986)0.635:0.16494000000000142,((('AT5G5260
0.1':1.27935,('AT5G14750.1':0.24953999999999965,('AT3G27920.1':0.5062300000000004,'AT5
G40330.1':0.26138000000000083)0.943:0.32048999999999995)0.989:0.6551999999999998)0.895:
0.35264000000000006,(('ZoMYB#10':0.9406600000000003,(('ZoMYB#185':0.04641999999999
946,'ZoMYB#184':0.012760000000000105)0.99:0.3192599999999999,('ZoMYB#87':0.05038000
0000000535,'ZoMYB#88':0.07249000000000017)0.995:0.3776299999999999)0.997:0.6448)0.88
3:0.2576300000000007,(((('AT1G66390.1':0.13771999999999984,'AT1G66380.1':0.18968999999
99997)0.57:0.028999999999999915,'AT1G56650.1':0.1962799999999998)0.687:0.08176999999
999968,'AT1G66370.1':0.28613)1.0:1.23569,('AT3G13540.1':0.78817,'ZoMYB#211':0.66901999
99999997)0.925:0.33010999999999946)0.87:0.27250000000000085)0.615:0.1418099999999995
5)0.35:0.2292399999999999,('AT5G35550.1':1.2428000000000008,((('ZoMYB#42':0.178620000
00000045,'ZoMYB#43':0.2833199999999998)0.966:0.3430800000000005,('ZoMYB#90':0.29474,
'ZoMYB#183':0.32608000000000015)0.973:0.3859900000000005)0.996:0.686869999999999,'Zo
MYB#116':0.9994199999999998)0.978:0.597570000000001)0.738:0.10687999999999942)0.967:
0.3061700000000007)0.852:0.11294999999999966)0.809:0.09984000000000037,(('ZoMYB#168
':1.1937600000000002,(('AT3G48920.1':0.96983,('AT4G25560.1':0.4033300000000004,'AT5G52
260.1':0.24099000000000004)0.987:0.4849800000000002)0.946:0.35189999999999966,('ZoMY
B#121':0.8086899999999995,('ZoMYB#85':0.2804000000000002,('ZoMYB#156':0.1799200000
0000008,'ZoMYB#196':0.25950000000000006)0.981:0.27357999999999993)0.997:0.60398)0.86
8:0.20847999999999978)0.948:0.41044000000000036)0.983:0.5931999999999995,((('AT5G266
60.1':0.6379199999999994,('ZoMYB#200':0.26762000000000086,('ZoMYB#107':0.1392699999
9999978,'ZoMYB#35':0.2918599999999998)0.946:0.19396000000000058)1.0:0.3866599999999
991)0.903:0.17617000000000083,('AT4G01680.1':0.7438399999999996,('ZoMYB#104':1.04819
99999999996,('AT1G09540.1':0.24892999999999965,'AT1G57560.1':0.3783599999999989)0.81:
0.19894000000000034)0.18:0.09446999999999939)0.211:0.15017000000000102)1.0:0.55098999
99999988,((('ZoMYB#44':0.5658599999999998,'ZoMYB#68':0.46232000000000006)0.998:0.78
32300000000005,('AT3G08500.1':0.6655100000000003,'AT5G12870.1':0.6224600000000002)0.
992:0.5678900000000002)0.816:0.1298300000000001,('AT1G63910.1':1.07191,('AT3G13890.1':
0.9913899999999991,('AT3G12720.1':0.5696199999999996,('ZoMYB#206':0.488850000000000
23,('ZoMYB#194':0.07458000000000009,'ZoMYB#101':0.19716000000000022)0.915:0.1530100
000000001)0.999:0.5475499999999993)0.698:0.21602999999999994)0.931:0.269740000000000
53)0.225:0.11265000000000036)0.896:0.18518999999999863)0.937:0.19231000000000087)0.95
5:0.2223000000000006)0.69:0.07784999999999975,((('AT5G56110.1':0.3125599999999995,('Zo
MYB#91':0.0,'ZoMYB#92':0.0):0.1762699999999997)0.999:1.2501700000000007,((('ZoMYB#1
13':1.1234599999999997,((((('ZoMYB#21':0.1698599999999999,'ZoMYB#8':0.26166000000000
09)0.998:0.4532199999999996,('ZoMYB#80':0.3857600000000003,'ZoMYB#190':0.14344)1.0:
0.8529100000000005)0.587:0.0955199999999996,'AT5G57620.1':0.5235200000000004)0.392:0.
08578000000000063,('ZoMYB#126':0.1617999999999995,'ZoMYB#52':0.1953800000000001)1.
0:0.786290000000001)0.587:0.06520999999999955,(('ZoMYB#26':0.1918299999999995,'ZoMY
B#1':0.7601499999999994)0.763:0.08244000000000007,('ZoMYB#195':0.43423000000000034,(
'AT4G37780.1':1.0631000000000004,('AT3G49690.1':0.18387000000000064,'AT5G65790.1':0.2

3709999999999987)0.998:0.4980600000000006)0.008:0.0965600000000002)0.827:0.152879999
99999968)0.888:0.1094900000000009)0.884:0.17222999999999988)0.308:0.082340000000000
3,((('ZoMYB#147':0.5133299999999998,'ZoMYB#153':0.47150999999999943)0.968:0.3921099
9999999974,('AT5G23000.1':0.9776999999999996,'AT2G36890.1':0.9054699999999993)0.406:0.
1149100000000007)0.833:0.15198,(('ZoMYB#9':0.20350999999999964,('ZoMYB#22':0.02649
9999999999524,'ZoMYB#12':0.015220000000000233)0.957:0.1468400000000008)1.0:0.57805
00000000002,(('ZoMYB#83':0.1596700000000002,'ZoMYB#189':0.09930000000000039)0.956:
0.11722999999999928,('ZoMYB#165':0.16597999999999935,'ZoMYB#54':0.289529999999999
2)0.446:0.0736699999999999)0.997:0.38463000000000047)0.96:0.2249799999999995)0.817:0.1
3211000000000084)1.0:0.7996199999999991,('AT1G18960.1':1.5491900000000003,('AT3G2847
0.1':0.40976999999999997,'ZoMYB#14':0.62547)0.97:0.39786000000000055)0.672:0.19442999
999999966)0.769:0.1395300000000006)0.859:0.13714000000000048,('AT1G74650.1':0.4292300
0000000044,((('AT1G08810.1':0.45536000000000065,('ZoMYB#108':0.28308,'ZoMYB#73':0.26
40199999999995)0.984:0.34526000000000057)0.999:0.5512299999999994,(((('ZoMYB#25':0.4
523399999999995,'ZoMYB#219':0.46432999999999947)0.957:0.14135000000000097,'ZoMYB#
45':0.6039600000000007)0.81:0.0411999999999999,((('ZoMYB#127':0.1854800000000001,'Zo
MYB#72':0.2841300000000002)0.982:0.20493000000000006,('ZoMYB#151':0.32734000000000
04,'ZoMYB#130':0.12399999999999967)0.997:0.28566)0.53:0.11296000000000017,'ZoMYB#17
2':0.5080999999999998)0.925:0.1142000000000003)0.961:0.11730000000000018,'AT3G28910.1
':0.4791800000000004)0.536:0.05966999999999967)0.62:0.052700000000000635,('AT3G47600.
1':0.16456999999999944,'AT5G62470.1':0.19051999999999936)1.0:0.28704000000000107)0.13
9:0.04321000000000019)1.0:0.8878000000000004)0.747:0.14278999999999975)0.726:0.118819
99999999948,(((('AT3G61250.1':0.5272999999999994,('ZoMYB#136':0.0371100000000002,'Zo
MYB#217':0.20612999999999992)0.988:0.34615999999999936)1.0:0.6606500000000004,(('Zo
MYB#66':0.17928999999999995,('ZoMYB#192':0.07598999999999911,'ZoMYB#99':0.0766799
9999999964)0.869:0.07711000000000023)1.0:0.6794200000000004,('AT3G01140.1':0.10055999
999999976,'AT5G15310.1':0.2319800000000008)0.993:0.4284600000000003)0.982:0.3780799
9999999975)0.892:0.32187000000000054,(((('ZoMYB#148':0.89175,('ZoMYB#70':0.941739999
9999994,'AT4G17785.1':0.8918400000000002)0.009:0.06230000000000466)0.504:0.10244999
999999926,((('AT5G10280.1':0.2368100000000002,'AT5G65230.1':0.19451000000000018)0.998:
0.3897199999999996,('AT1G34670.1':0.5521199999999995,(('ZoMYB#218':0.331879999999999
995,('ZoMYB#7':0.1257199999999994,('ZoMYB#13':0.012559999999999683,'ZoMYB#20':0.00
9570000000000078)0.999:0.10749000000000031)0.73:0.07087999999999983)0.909:0.08762999
999999987,('ZoMYB#58':0.37448999999999977,'ZoMYB#64':0.5041099999999998)0.708:0.088
3500000000000015)0.985:0.21126000000000023)0.096:0.10401999999999934)0.999:0.544579999
9999998,('AT3G02940.1':0.17709000000000064,'AT5G16770.1':0.08225999999999978)1.0:0.64
8969999999999994)0.495:0.10975000000000001)0.977:0.2556900000000013,(('AT4G28110.1':0.7
082699999999997,'AT5G54230.1':0.8768099999999999)0.17:0.14563999999999933,('ZoMYB#
17':0.9880400000000007,(('AT4G21440.1':0.23105999999999938,'AT4G05100.1':0.44052999999
999987)1.0:0.40163999999999955,('ZoMYB#118':0.16892999999999958,'ZoMYB#53':0.219319
99999999974)1.0:0.46126999999999985)0.954:0.19914000000000076)0.334:0.04905999999999
899)0.963:0.19370000000000154)0.546:0.08942999999999834,(('AT5G14340.1':0.64407000000
00001,('ZoMYB#19':0.05935000000000236,'ZoMYB#4':0.3774899999999999)1.0:0.63246)0.9
76:0.3889300000000002,((('AT1G66230.1':0.4560800000000004,(('AT4G22680.1':0.372790000

0000002,'AT4G12350.1':0.33966999999999999)0.989:0.34652999999999956,('ZoMYB#125':0.56
39900000000004,'ZoMYB#94':0.20162999999999975)0.942:0.34482999999999997)0.659:0.128
7199999999995)0.9:0.21968999999999994,('ZoMYB#224':0.68161,('AT5G16600.1':0.59272999
99999995,(('ZoMYB#71':0.30690999999999935,'ZoMYB#178':0.32922999999999999)0.942:0.176
98999999999998,'ZoMYB#67':0.37243999999999992)0.457:0.08445000000000036)0.768:0.0798
1999999999978)0.909:0.19402999999999997)0.803:0.10184000000000104,'AT5G62320.1':1.155
8400000000004)0.384:0.22541999999999973)0.999:0.53797999999999992)0.407:0.07523000000
000124)0.291:0.09267000000000003,(((('AT3G23250.1':0.62383999999999995,(('ZoMYB#142':0.
000840,('ZoMYB#141':0.000830,(('ZoMYB#138':0.0,'ZoMYB#140':0.0):0.03786999999999985,'
ZoMYB#139':0.03786999999999985)0.822:0.03777999999999997)0.905:0.07586000000000048)
0.952:0.23091999999999935,'ZoMYB#27':0.27748999999999935)1.0:0.46326)0.602:0.16049000
000000024,('AT1G06180.1':0.40001999999999996,'AT2G31180.1':0.32548999999999994)0.988:0.
32303000000000015)0.93:0.20141000000000001,(('ZoMYB#76':0.83658999999999993,('ZoMYB#
75':0.63525000000000001,'ZoMYB#198':0.72634000000000004)0.911:0.30936999999999995)0.163:
0.10057000000000027,('ZoMYB#74':0.41536000000000006,'ZoMYB#146':0.58793000000000001)
0.96:0.30066999999999993)0.818:0.22994000000000003)0.884:0.16131999999999999,(('ZoMYB#
51':0.88884000000000001,(('AT1G16490.1':0.23191999999999968,'AT1G79180.1':0.22328999999
999954)0.98:0.43266999999999999,('AT1G56160.1':0.53478000000000005,'AT3G12820.1':0.5877
600000000003)0.901:0.28248999999999924)0.997:0.78269000000000006)0.499:0.199709999999
9996,('ZoMYB#145':0.76243000000000002,'ZoMYB#39':1.10738)0.962:0.37340999999999998)0.
829:0.16328000000000003)0.994:0.40571000000000009)0.621:0.09554999999999936);
